# Cuproptosis-Related Gene *DLAT* as a Novel Biomarker Correlated with Prognosis, Chemoresistance, and Immune Infiltration in Pancreatic Adenocarcinoma: A Preliminary Study Based on Bioinformatics Analysis

**DOI:** 10.3390/curroncol30030228

**Published:** 2023-03-02

**Authors:** Zengli Fang, Wei Wang, Yuan Liu, Jie Hua, Chen Liang, Jiang Liu, Bo Zhang, Si Shi, Xianjun Yu, Qingcai Meng, Jin Xu

**Affiliations:** 1 Department of Pancreatic Surgery, Fudan University Shanghai Cancer Center, No. 270 Dong’An Road, Xuhui District, Shanghai 200032, China; 2 Department of Oncology, Shanghai Medical College, Fudan University, Shanghai 200032, China; 3 Shanghai Pancreatic Cancer Institute, Shanghai 200032, China; 4 Pancreatic Cancer Institute, Fudan University, Shanghai 200032, China; 5 Department of Endoscopy, Fudan University Shanghai Cancer Center, Shanghai 200032, China

**Keywords:** cuproptosis, *DLAT*, bioinformatics, prognosis, chemoresistance, immune infiltration, biomarker, pancreatic adenocarcinoma

## Abstract

A novel form of cell death, cuproptosis, was recently identified to be mediated by the binding of copper to lipoylated enzymes of the tricarboxylic acid cycle. Cuproptosis-related genes (CRGs) may play a crucial role in the progression of pancreatic adenocarcinoma (PAAD), which often exhibits metabolic reprogramming. In the present study, univariate Cox regression analysis and Kaplan–Meier survival analysis were performed to identify prognostic CRGs. Data from the Cancer Therapeutics Response Portal and the Genomics of Drug Sensitivity in Cancer database were downloaded for drug sensitivity analysis. *DLAT* was identified as the only prognostic CRG in PAAD (HR = 2.72; 95% CI, 1.10–6.74). Functional enrichment analyses indicated that the basic function of *DLAT* is closely related to metabolism, and multiple tumor-promoting and immune response-related pathways were enriched in *DLAT*-high PAAD samples. The influence of *DLAT* and related genes on cancer immunity was evaluated by comprehensive immune infiltration analyses, which revealed the value of these genes as biomarkers for evaluating the sensitivity to immunotherapy. Additionally, high *DLAT* expression induced drug resistance, and significantly increased resistance to commonly used chemotherapeutics in PAAD, such as gemcitabine, oxaliplatin, 5-fluorouracil, and irinotecan. In conclusion, our study preliminarily revealed the prognostic value of *DLAT*, which is correlated with PAAD progression, chemoresistance, and immune infiltration, providing a valuable reference for PAAD treatment. However, our findings need to be confirmed by further in vivo and in vitro experiments.

## 1. Introduction

Pancreatic adenocarcinoma (PAAD) is one of the most lethal malignancies, with a 5-year survival rate of less than 10% [1]. In the United States, the third leading cause of cancer-related mortality is PAAD, of which the incidence increases by 0.5% to 1.0% per year [2,3]. Surgical resection is the first choice for the treatment of resectable PAAD. After surgery, appropriate adjuvant chemotherapy, including modified FOLFIRINOX or gemcitabine-based regimens, should be applied according to the patient’s functional status [4]. In other cases, newly diagnosed patients may have developed to borderline resectable or unresectable PAAD, and neoadjuvant chemotherapy or comprehensive treatment are adopted [4]. The dismal prognosis of PAAD is closely related to its highly malignant nature and therapeutic resistance [4,5]. Therefore, for the welfare of PAAD patients, studies identifying new prognostic markers, clarifying the mechanism of therapy resistance, and developing innovative treatment methods are urgently needed.

As a cofactor of many essential enzymes, copper plays a crucial role in various biological processes [6,7]. However, the accumulation of free intracellular copper induces cytotoxicity, so normal cells maintain low levels of copper through a set of molecular mechanisms [8]. Several studies have pointed out that the copper concentration in various malignant tumors is higher than that in normal tissues and may have an impact on malignant biological behavior [9,10]. A recent study identified a unique form of copper-dependent cell death, termed cuproptosis, that is closely related to ferredoxin 1 (FDX1), which has a role in regulating protein lipoylation [11,12]. Further mechanistic exploration showed that copper directly binds to lipoylated tricarboxylic acid (TCA) enzymes, leading to the oligomerization of lipoylated proteins, resulting in acute proteotoxic stress and eventually cuproptosis [12]. Because of the higher content of lipoylated TCA enzymes, cells actively undergoing the TCA cycle are more susceptible to cuproptosis. Metabolic reprogramming is one of the typical hallmarks of PAAD, so cuproptosis-related genes (CRGs) may provide new prognostic markers and guide the development of new therapeutic regimens.

**Figure 1:**
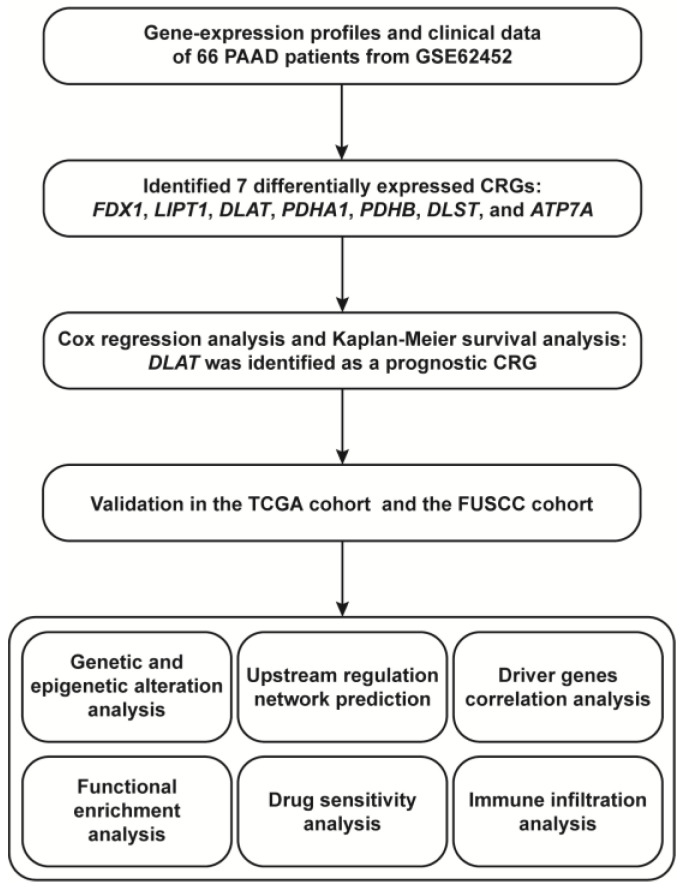
The flow chart of the present study.

In the present study, we identified *DLAT* as a prognostic CRG based on bioinformatics analysis. We then analyzed the genetic and epigenetic alterations of *DLAT* and predicted the regulatory transcription factors (TFs) and microRNAs (miRNAs). To further elucidate the biological function of *DLAT* and its role in PAAD, we constructed protein–protein interaction (PPI) networks and performed functional enrichment analyses. Drug sensitivity analysis and evaluation of immune infiltration were also conducted to investigate the guiding significance of *DLAT* in PAAD treatment. The flow chart of the present study is shown in Figure 1.

## 2. Materials and Methods

### 2.1. Data Acquisition and Processing

Gene-expression profiles and clinical information of PAAD samples used for the training cohort were acquired from the GSE62452 dataset in the Gene Expression Omnibus (GEO) database (https://www.ncbi.nlm.nih.gov/geo/ (accessed on 25 August 2022)). RNA-sequencing (RNA-Seq) data and corresponding clinical data of PAAD samples obtained from The Cancer Genome Atlas (TCGA) database (https://cancergenome.nih.gov/ (accessed on 25 August 2022)) were used as the validation cohort. The Genotype-Tissue Expression (GTEx) database (https://commonfund.nih.gov/GTEx/ (accessed on 25 August 2022)) was used to acquire RNA-Seq data of normal pancreas tissue samples. The protein expression profiles of PAAD samples were downloaded from The National Cancer Institute’s Clinical Proteomic Tumor Analysis Consortium (CPTAC) database (https://cptac-data-portal.georgetown.edu/ (accessed on 25 August 2022)). The list of CRGs was acquired from the previous literature [12], and the list of immunomodulatory genes (Table S1) was acquired from the TISIDB database [13].

### 2.2. Identification of Prognostic CRGs

First, univariate Cox regression analysis and Kaplan–Meier (KM) survival analysis were performed to identify prognostic CRGs in the training cohort, and *p* < 0.05 was considered to indicate statistical significance. Subsequently, the prognostic CRGs were validated in validation cohorts.

### 2.3. Quantitative Real-Time PCR (qRT-PCR)

A total of 85 PAAD tissues and 18 adjacent nontumor tissues (paired with tumor tissues) were collected from patients who underwent surgical resection at Fudan University Shanghai Cancer Center (FUSCC), between 2016 and 2017. Total RNA was isolated from PAAD tissues with TRIzol reagent (Invitrogen, Carlsbad, CA, USA) and subsequently reverse transcribed into cDNA using a PrimeScript RT Reagent Kit (TaKaRa, Tokyo, Japan) according to the manufacturer’s instructions. The expression of candidate genes was determined using an ABI 7900HT Real-Time PCR System (Applied Biosystems, Foster City, CA, USA). The 2^-ΔΔCt^ method was applied to calculate the relative expression levels of target genes. The primer sequences used in the present study are shown below:

*DLAT*: Forward 5′-CGGAACTCCACGAGTGACC-3′,

Reverse 5′-CCCCGCCATACCCTGTAGT-3′.

*GAPDH*: Forward 5′-GGAGCGAGATCCCTCCAAAAT-3′,

Reverse 5′-GGCTGTTGTCATACTTCTCATGG-3′.

### 2.4. Western Blotting

Western blotting was performed as described in our previous study [14]. The following antibodies were used in the present study: anti-DLAT (1:2000, 13426-1-AP) and anti-GAPDH (1:30,000, 60004-1-Ig) antibodies, which were purchased from Proteintech (Chicago, IL, USA).

### 2.5. Immunofluorescence

Formalin-fixed and paraffin-embedded PAAD tissue samples were divided into 5 μm thick sections for immunofluorescence. After being deparaffinized and hydrated, sections were incubated overnight with anti-DLAT antibody (1:200, 13426-1-AP) at 4 °C. After PBS washing, fluorescently labeled secondary antibody was added and incubated against light. Ultimately, cell nuclei were counterstained blue with DAPI. Images were obtained with a fluorescence microscope (Olympus, Tokyo, Japan).

### 2.6. Analyses of Genetic and Epigenetic Alterations

Information on genetic alterations of TCGA-PAAD samples was obtained from the cBioPortal database (http://www.cBioPortal.org/ (accessed on 25 August 2022)) and analyzed and visualized with online instructions. DNA methylation data for TCGA-PAAD samples were downloaded from the MethSurv database [15].

### 2.7. Prediction of TFs and miRNAs

Network Analysis is a database that integrates the existing regulations in multiple databases and the potential regulatory relationship based on TF binding sites [16]. In the present study, the TFs and miRNAs potentially regulating *DLAT* were predicted and visualized by Network Analysis.

### 2.8. Functional Enrichment Analysis

Proteins that interact or are coexpressed or colocalized with DLAT were identified using the STRING database (https://cn.string-db.org/ (accessed on 25 August 2022)) and the GeneMANIA database (http://genemania.org/ (accessed on 25 August 2022)), with nodes set to 30 per database. The coding genes of all proteins present in the nodes formed a gene set for Gene Ontology (GO) [17] and Kyoto Encyclopedia of Genes and Genomes (KEGG) [18] pathway analyses to investigate the basic function of *DLAT*. The LinkedOmics database [19] was used to screen the genes that significantly correlated with *DLAT* expression in TCGA-PAAD samples. The genes with |r| > 0.5 and *p* < 0.05 formed a gene set, which was used for GO and KEGG pathway analyses to determine the potential biological function of *DLAT* in PAAD. The PAAD samples were equally divided into two groups according to the expression of *DLAT* for Gene Set Enrichment Analysis (GSEA) [20]. GO and KEGG pathway analyses were performed using the R package “clusterProfiler” (version 3.18.1, pvalueCutoff = 0.05, qvalueCutoff = 0.05, readable = TRUE) [21]. GSEA was performed via the GSEA 4.1.0 program according to the “h.all.v7.5.symbols.gmt” and “c2.cp.kegg.v7.5.symbols.gmt” gene sets, with thresholds of normalized *p* < 0.05 and false discovery rate (FDR) < 0.25 considered to indicate significant enrichment.

### 2.9. Drug Sensitivity Analysis

The Cancer Therapeutics Response Portal (CTRP) (https://portals.broadinstitute.org/ctrp.v2.1/ (accessed on 25 August 2022)) was used to evaluate the correlation between the half-maximal inhibitory concentration (IC50) and *DLAT* expression of PAAD cell lines. The IC50 values of medication to TCGA-PAAD samples were calculated using the R package “pRRophetic” (version 0.5, batchCorrect = ‘eb’, powerTransformPhenotype = T, removeLowVaryingGenes = 0.2, minNumSamples = 10) [22] based on the data acquired from CTRP and the Genomics of Drug Sensitivity in Cancer (GDSC) database (https://www.cancerrxgene.org/ (accessed on 25 August 2022)) to estimate the effect of *DLAT* in PAAD on drug sensitivity.

### 2.10. Evaluation of Immune Infiltration

First, the Tumor IMmune Estimation Resource (TIMER) database [23] was used to evaluate the correlation between the expression of *DLAT* and related key genes and the tumor microenvironmental infiltration abundance of tumor-infiltrating immune cells (TIICs), including B cells, CD8+ T cells, CD4+ T cells, macrophages, neutrophils, and dendritic cells. Subsequently, correlations between these genes and the expression of immunomodulatory genes were analyzed and presented. The ESTIMATE algorithm was used to calculate the StromalScore (SS), ImmuneScore (IS), and ESTIMATEScore (ES) for PAAD samples [24]. The PAAD samples were equally divided into two groups according to the expression of *DLAT* and related key genes, and the differences in SS, IS, and ES between the two groups were assessed. Genes with a strong association (|r| > 0.4, *p* < 0.05) with a high number of immunomodulatory genes, as well as genes significantly affecting SS, IS, and ES, were identified as key immune-related genes. Single-sample Gene Set Enrichment Analysis (ssGSEA) using the R package “GSVA” (version 1.38.2, method = ‘ssgsea’, kcdf = ‘Gaussian’, abs.ranking = TRUE) [25,26] and the CIBERSORT algorithm [27] were performed to evaluate correlations between key immune-related genes and the infiltration abundance of TIICs. Finally, the GEPIA2021 database [28] was used to explore the expression of *DLAT* and related key genes in TIICs.

### 2.11. Statistical Analysis

R 4.1.1 and GraphPad Prism 9.3.1 software were the main tools used to conduct the statistical analysis and visualization. Correlation coefficients were calculated by Pearson’s or Spearman’s correlation analysis according to data type. For comparisons between two groups, Student’s t test and Wilcoxon’s t test were chosen as parametric and nonparametric methods, respectively. For comparisons among more than two groups, for data with a normal distribution, one-way analysis of variance was used; otherwise, the Kruskal–Wallis test was performed. The log-rank test was performed to evaluate the differences in the KM survival analyses. A *p* value < 0.05 was considered to indicate a statistically significant difference.

## 3. Results

### 3.1. DLAT Was Identified as a Prognostic CRG

**Figure 2:**
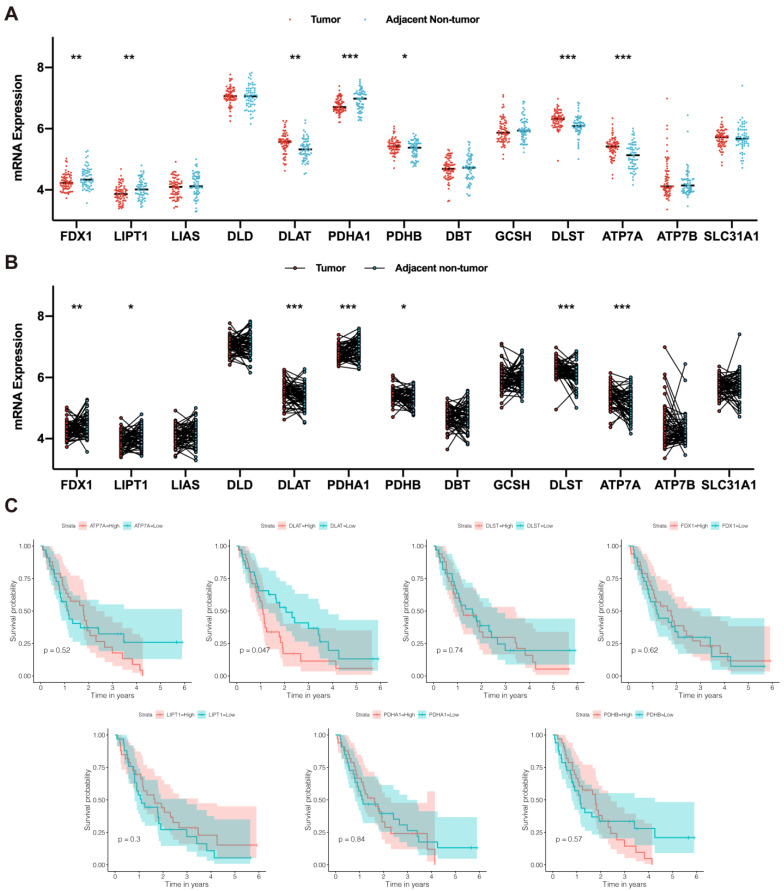
*DLAT* was identified as a prognostic CRG. (**A**) The difference in CRG expression between tumor and nontumor tissue samples: *FDX1*, *LIPT1*, *DLAT*, *PDHA1*, *PDHB*, *DLST*, and *ATP7A* were significantly differentially expressed; (**B**) the difference in CRG expression in paired tumor and adjacent nontumor tissue samples: *FDX1*, *LIPT1*, *DLAT*, *PDHA1*, *PDHB*, *DLST*, and *ATP7A* were significantly differentially expressed; (**C**) Kaplan–Meier survival curves revealed the prognostic value of *FDX1*, *LIPT1*, *DLAT*, *PDHA1*, *PDHB*, *DLST*, and *ATP7A* (* *p* value < 0.05; ** *p* value < 0.01; *** *p* value < 0.001).

Based on the study performed by Tsvetkov et al. [12], we screened 13 CRGs, including *FDX1*, *LIPT1*, *LIAS*, *DLD*, *DLAT*, *PDHA1*, *PDHB*, *DBT*, *GCSH*, *DLST*, *ATP7A*, *ATP7B*, and *SLC31A1*. According to the gene-expression profiles acquired from the GSE62452 dataset, we compared the expression of these 13 CRGs in tumor and nontumor tissues of PAAD patients. We found that *FDX1*, *LIPT1*, *DLAT*, *PDHA1*, *PDHB*, *DLST*, and *ATP7A* were significantly differentially expressed in tumor and nontumor tissues (Figure 2A), with the standard of *p* < 0.05. The differential expression of these genes was verified in paired tissue samples (Figure 2B). Next, univariate Cox regression analysis (Table 1) and KM survival analysis (Figure 2C) were performed to assess the association between the expression of these genes and overall survival in PAAD patients. Among these genes, only *DLAT* had a significant prognostic value.

**Figure 3:**
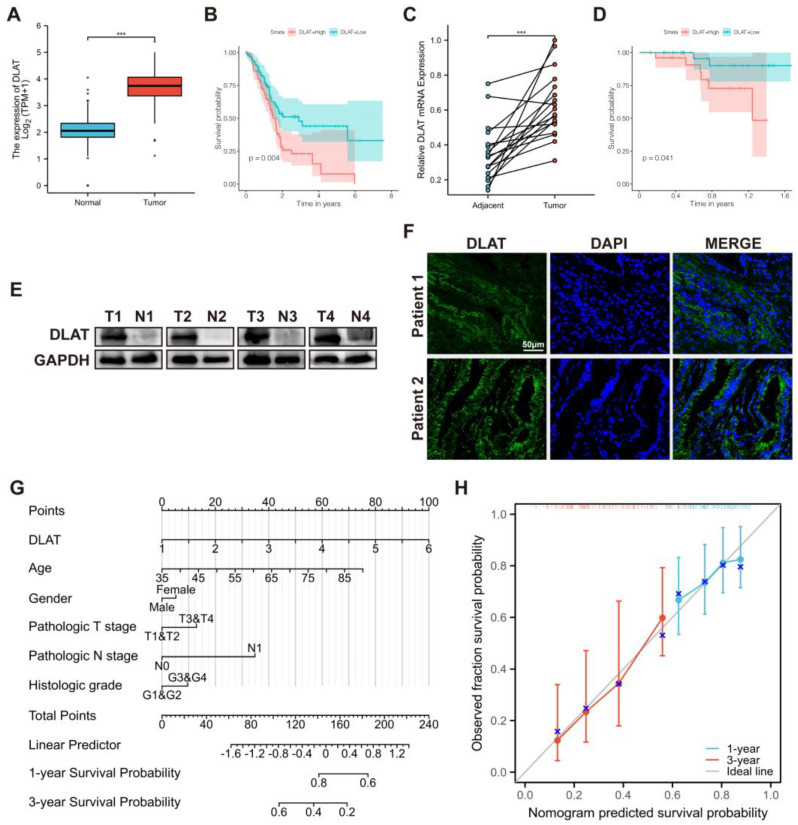
The expression and prognostic value of *DLAT* was validated in the TCGA cohort and the FUSCC cohort. (**A**) The differential expression of *DLAT* in tumor and nontumor tissue samples was validated by analysis of the TCGA database and the GTEx database; (**B**) the role of *DLAT* in predicting the overall survival of patients with PAAD was validated by the TCGA cohort; (**C**) qRT–PCR confirmed that the expression of *DLAT* in tumor tissues is significantly higher than that in paired adjacent nontumor tissues; (**D**) the role of *DLAT* in predicting the overall survival of patients with PAAD was validated by the FUSCC cohort; (**E**) Western blotting confirmed that the expression of DLAT in tumor tissues is higher than that in paired adjacent nontumor tissues (the whole western blots are shown in Figure S3); (**F**) tissue immunofluorescence showed the high expression of DLAT in PAAD; (**G**,**H**) *DLAT*–related nomogram and calibration curve for survival probability prediction (*** *p* value < 0.001).

The differential expression of *DLAT* in tumor and nontumor tissue samples was validated by analysis of the TCGA database and the GTEx database (Figure 3A). Based on the data from TCGA, the prognostic effect of *DLAT* was tested (Figure 3B). To verify the abnormal expression of *DLAT* more convincingly, we detected the expression level of *DLAT* in tumor and adjacent nontumor tissues from 18 PAAD patients using qRT-PCR. As shown in Figure 3C, the expression of *DLAT* in tumor tissues is significantly higher than that in paired adjacent nontumor tissues. Then, we isolated proteins from four pairs of tumor and adjacent tissues and verified the high expression of DLAT in PAAD at the protein level (Figure 3E). The results of tissue immunofluorescence also showed the high expression of DLAT in PAAD (Figure 3F). Based on the follow-up of 67 patients with PAAD, we constructed the FUSCC cohort and again validated the prognostic value of *DLAT* (Figure 3D). Additionally, we constructed a *DLAT*-related nomogram with clinicopathological characteristics for survival probability prediction (Figure 3G,H).

### 3.2. Analyses of Genetic and Epigenetic Alterations

**Figure 4:**
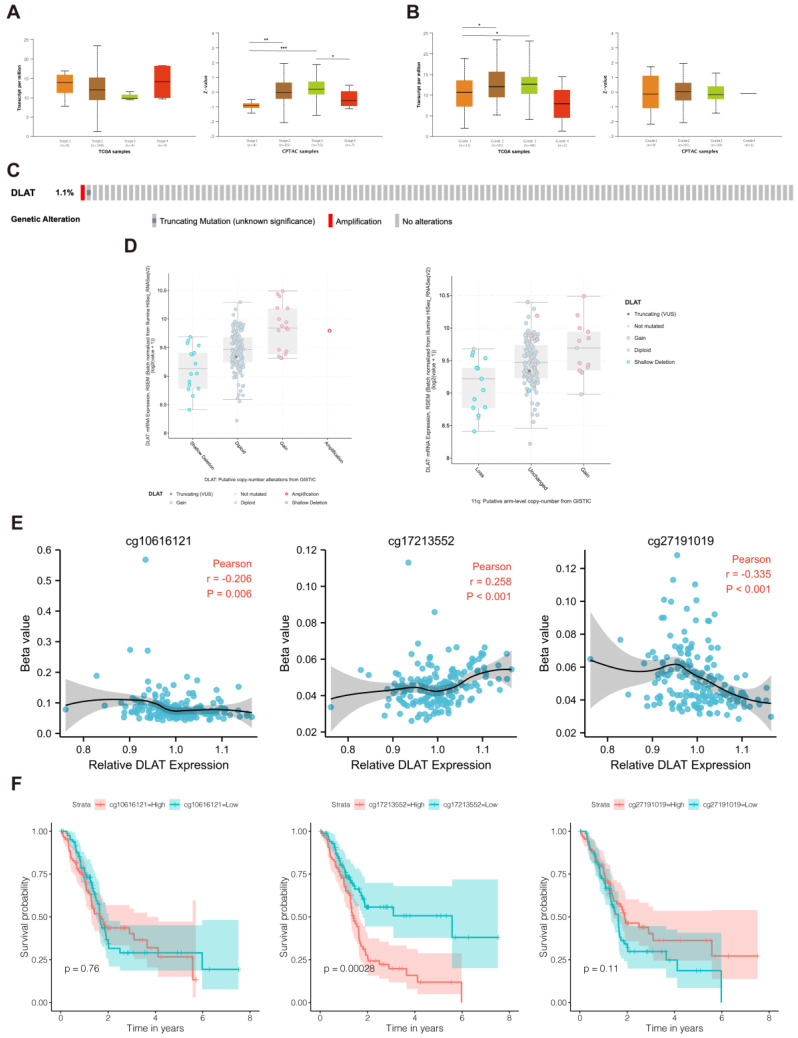
Analyses of genetic and epigenetic alterations. (**A**) mRNA and protein expression of DLAT in different pathological stages; (**B**) mRNA and protein expression of DLAT in different histologic grades; (**C**) OncoPrint presented the genetic alterations of *DLAT* in TCGA–PAAD samples; (**D**) *DLAT* expression in different conditions of copy number variations and 11q arm-level copy number alterations; © correlation between *DLAT* expression and the methylation of cg10616121, cg17213552, and cg27191019; (**F**) KM survival curves revealed the prognostic value of the methylation of cg10616121, cg17213552, and cg27191019 (* *p* value < 0.05; ** *p* value < 0.01; *** *p* value < 0.001).

To explore the factors that influence *DLAT* expression in PAAD, we analyzed the potential relationship between *DLAT* expression and the clinical characteristics of patients. As shown in Figure 4A,B, *DLAT* expression was significantly different among patients grouped by tumor distinct stage and histologic grade but did not differ among patients grouped by other clinical characteristics, such as age and sex. Subsequently, we analyzed the genetic alterations of *DLAT* in TCGA-PAAD samples based on the cBioPortal database. One sample presented a truncating mutation, and another sample presented genetic amplification of *DLAT* (Figure 4C). Then, we analyzed differences in gene expression levels between different types of copy number variations (CNVs), confirming that *DLAT* expression is affected by CNVs and 11q arm-level copy number alterations (Figure 4D). In addition, the correlation between *DLAT* expression and DNA methylation was analyzed. The methylation of cg10616121, cg17213552, and cg27191019 was significantly correlated with *DLAT* gene expression (Figure 4E), and patients with higher methylation of cg17213552 had a worse prognosis (Figure 4F).

### 3.3. Prediction of TFs and miRNAs Potentially Regulating DLAT

**Figure 5:**
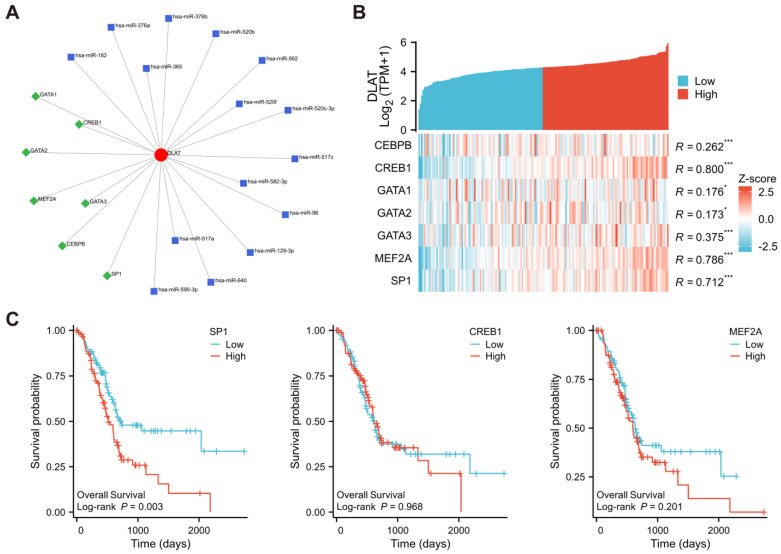
Prediction of TFs and miRNAs potentially regulating *DLAT*. (**A**) TFs and miRNAs predicted to potentially regulate *DLAT* according to Network Analysis; (**B**) the correlation between the expression *DLAT* and the expression of predicted TFs: the expression of *CREB1*, *MEF2A*, and *SP1* had a strong correlation; (**C**) KM survival curves revealed the prognostic value of *SP1*, *CREB1*, and *MEF2A* (* *p* value < 0.05; *** *p* value < 0.001).

To further verify the factors affecting *DLAT* expression in PAAD, the TFs and miRNAs potentially regulating *DLAT* were predicted and visualized by Network Analysis (Figure 5A). According to the RNA-Seq data from TCGA-PAAD samples, the correlation between the expression of *DLAT* and TFs predicted to act on *DLAT* was calculated. The expression of *CREB1*, *MEF2A*, and *SP1* had a strong correlation (|r| > 0.4, *p* < 0.05) with the expression of *DLAT* (Figure 5B), and *SP1* had a prognostic effect (Figure 5C).

### 3.4. Correlations between DLAT and Major Driver Genes in PAAD

**Figure 6:**
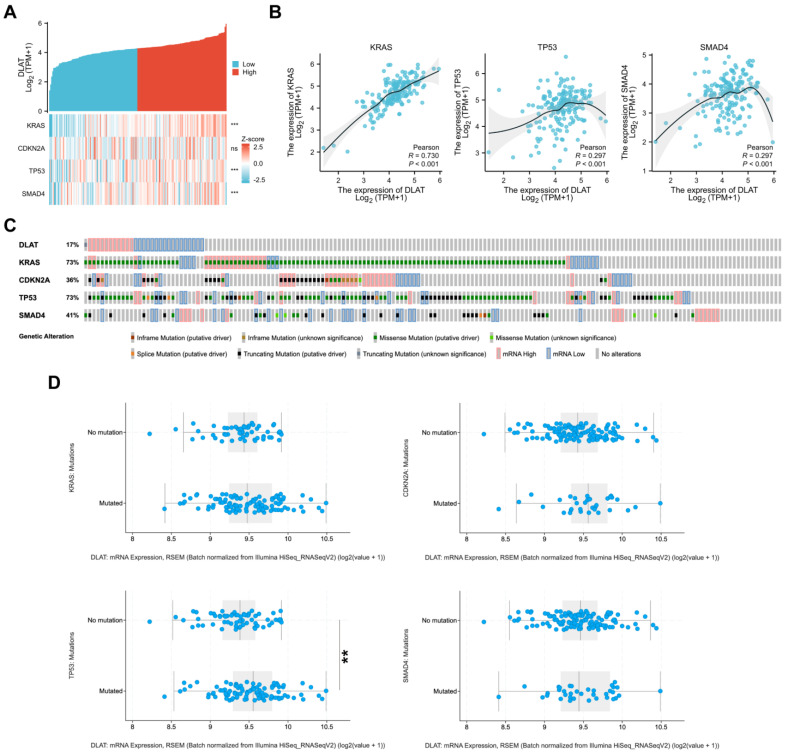
Correlations between *DLAT* and major driver genes in PAAD. (**A**,**B**) Correlation analysis shows that there are significant correlations between the expression of *DLAT* and *KARS* (r = 0.730), *TP53* (r = 0.297), and *SMAD4* (r = 0.297); (**C**) OncoPrint presented the mutation patterns of *KARS*, *CDKN2A*, *TP53*, and *SMAD4* and mRNA expression of *DLAT* in TCGA–PAAD samples; (**D**) the difference in the expression of *DLAT* between driver gene-mutated groups and no mutation groups (** *p* value < 0.01; *** *p* value < 0.001).

Previous studies have shown that *KARS*, *CDKN2A*, *TP53*, and *SMAD4* serve as major driver genes in the initiation and progression of PAAD. To further explore the role of *DLAT* in pancreatic tumorigenesis, we analyzed the correlation of *DLAT* with driver gene expression in TCGA-PAAD samples. As shown in Figure 6A,B, there are significant correlations between the expression of *DLAT* and *KARS* (r = 0.730, *p* < 0.001), *TP53* (r = 0.297, *p* < 0.001), and *SMAD4* (r = 0.297, *p* < 0.001). Gene variants in *KARS*, *CDKN2A*, *TP53*, and *SMAD4* are crucial events in pancreatic tumorigenesis. Next, we further analyzed the correlation between *DLAT* and the mutation patterns of driver genes. We used the cBioPortal database to generate OncoPrint to display the mutation patterns of *KARS*, *CDKN2A*, *TP53*, and *SMAD4* and mRNA expression (set the z-score threshold to 1.2 to define mRNA High and mRNA Low) of *DLAT* in TCGA-PAAD samples. The mutation rate of *KRAS* and *TP53* in patients with significantly high expression of *DLAT* was 100%, while the mutation rate of patients with significantly low expression of *DLAT* was only 64.7% and 47.1%, respectively (Figure 6C). We then divided the samples into two groups according to the mutation status and found that there was a significant difference in the expression level of *DLAT* between the *TP53*-mutated group and no mutation group (Figure 6D).

### 3.5. Functional Enrichment Analysis

**Figure 7:**
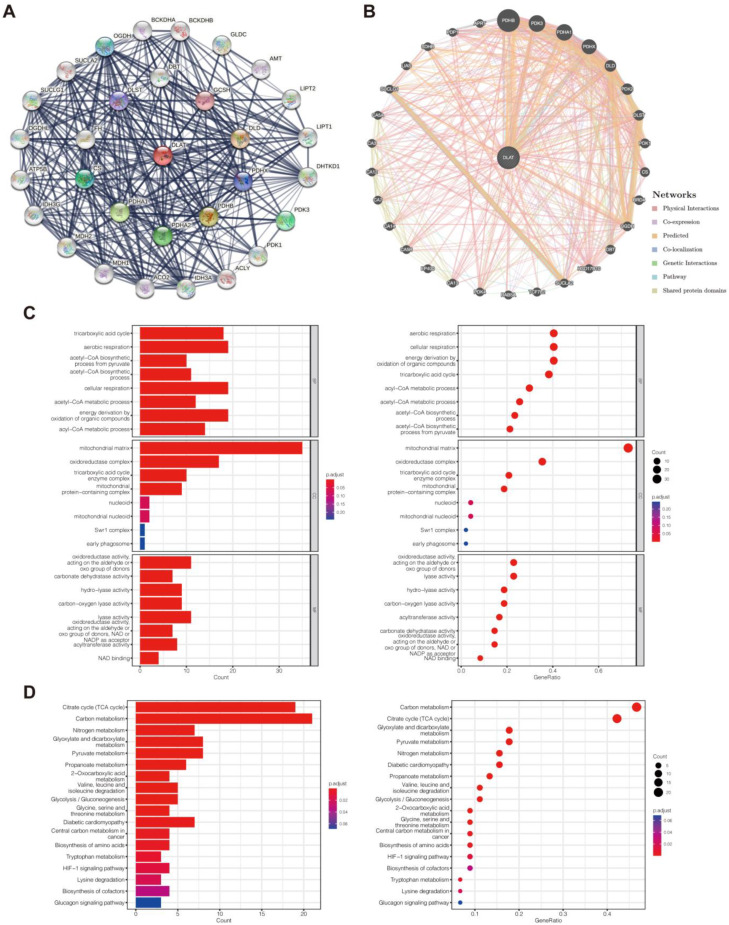
Functional enrichment analysis for studying the basic biological function of *DLAT*. (**A**) DLAT–related PPI networks constructed with the STRING database; (**B**) DLAT–related PPI networks constructed with the GeneMANIA database; (**C**) the results of GO analysis based on the gene set constructed by the genes presented in the two PPI networks; (**D**) the results of KEGG pathway analysis based on the gene set constructed by the genes presented in the two PPI networks.

**Figure 8:**
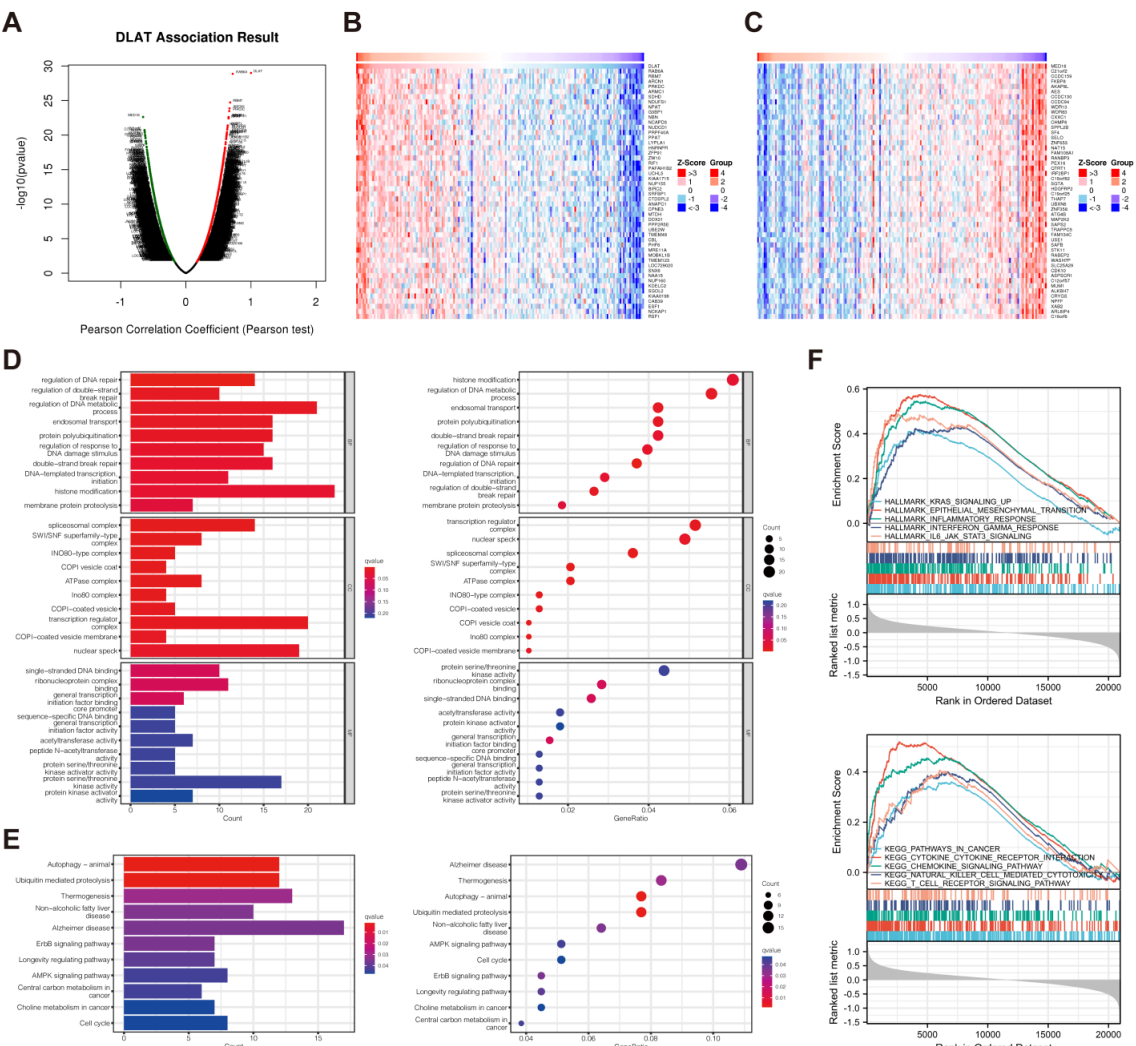
Functional enrichment analysis for investigating the potential biological function of *DLAT* in PAAD. (**A**) Genes significantly correlated with *DLAT* in PAAD identified with LinkedOmics; (**B**,**C**) heatmap presenting the top 50 genes positively and negatively coexpressed with *DLAT*; (**D**) the results of GO analysis based on the gene set constructed by the genes with a significant correlation with *DLAT*; (**E**) the results of KEGG pathway analysis based on the gene set constructed by the genes with a significant correlation with *DLAT*; (**F**) the results of GSEA.

To study the basic biological function of *DLAT*, we identified the genes closely correlated with *DLAT* based on the STRING database and the GeneMANIA database (Figure 7A,B). The genes presented in the two networks were used to construct a gene set for GO (consisting of biological process (BP), cell composition (CC), and molecular function (MF) categories) and KEGG pathway analyses (Figure 7C,D). In the KEGG analysis, the five most significantly enriched functions were the TCA cycle, carbon metabolism, nitrogen metabolism, glyoxylate and dicarboxylate metabolism, and pyruvate metabolism. To investigate the potential biological function of *DLAT* in PAAD, we used the LinkedOmics database to identify the genes with a significant correlation (|r| > 0.5, *p* < 0.05) with *DLAT* (Figure 8A–C) according to the RNA-Seq data from TCGA-PAAD samples to form a gene set for GO and KEGG pathway analyses (Figure 8D,E). The TCGA-PAAD samples were equally divided into two groups according to the expression of *DLAT* for GSEA (Figure 8F, Tables S2 and S3). In the KEGG analysis, significantly enriched functions included autophagy-animal, ubiquitin mediated proteolysis, ErbB signaling pathway, AMPK signaling pathway, central carbon metabolism in cancer, choline metabolism in cancer, etc. In GSEA, HALLMARK features that are significantly enriched included KRAS_SIGNALING_UP, EPITHELIAL_MESENCHYMAL_TRANSITION, INFLAMMATORY_RESPONSE, INTERFERON_GAMMA_RESPONSE, IL6_JAK_STAT3_SIGNALING, etc.; significantly enriched KEGG features included PATHWAYS_IN_CANCER, CYTOKINE_CYTOKINE_RECEPTOR_INTERACTION, CHEMOKINE_SIGNALING_PATHWAY, NATURAL_KILLER_CELL_MEDIATED_CYTOTOXICITY, T_CELL_RECEPTOR_SIGNALING_PATHWAY, etc.

### 3.6. Drug Sensitivity Analysis

**Figure 9:**
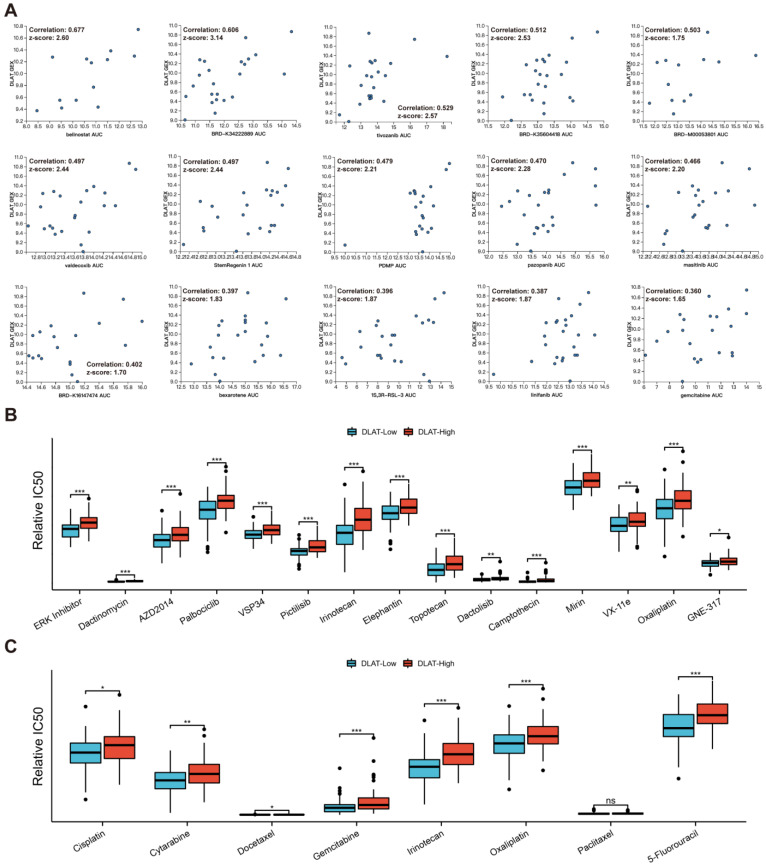
Drug sensitivity analysis revealed the effect of *DLAT* on therapeutic resistance. (**A**) The correlation between *DLAT* expression and the responses of PAAD cell lines to the top 15 influenced drugs; (**B**) the difference in the IC50s of the top 15 drugs whose sensitivity was most affected by *DLAT* between *DLAT*—high and *DLAT*—low PAAD samples; (**C**) the difference in the IC50s of the drugs commonly used for digestive system tumors between *DLAT*—high and *DLAT*—low PAAD samples (* *p* value < 0.05; ** *p* value < 0.01; *** *p* value < 0.001).

We first analyzed the effect of *DLAT* on drug sensitivity at the cellular level. Based on CTRP, we found that the expression level of *DLAT* significantly affected the resistance of PAAD cell lines to multiple drugs, and Figure 9A shows the 15 most influential drugs. Furthermore, based on data from the GDSC and CTRP, we analyzed the correlation between the IC50 values of compounds and *DLAT* expression in PAAD samples (Tables S4 and S5). Figure 9B presents the 15 drugs whose sensitivity was most affected by *DLAT* and the difference in the IC50s of these drugs between *DLAT*-high and *DLAT*-low samples. Cisplatin, cytarabine, docetaxel, gemcitabine, irinotecan, oxaliplatin, paclitaxel, and 5-fluorouracil are commonly used chemotherapeutic agents for digestive system tumors, and PAAD samples with high *DLAT* expression showed lower sensitivity to these drugs than those with low *DLAT* expression (Figure 9C).

### 3.7. Evaluation of Immune Infiltration

**Figure 10:**
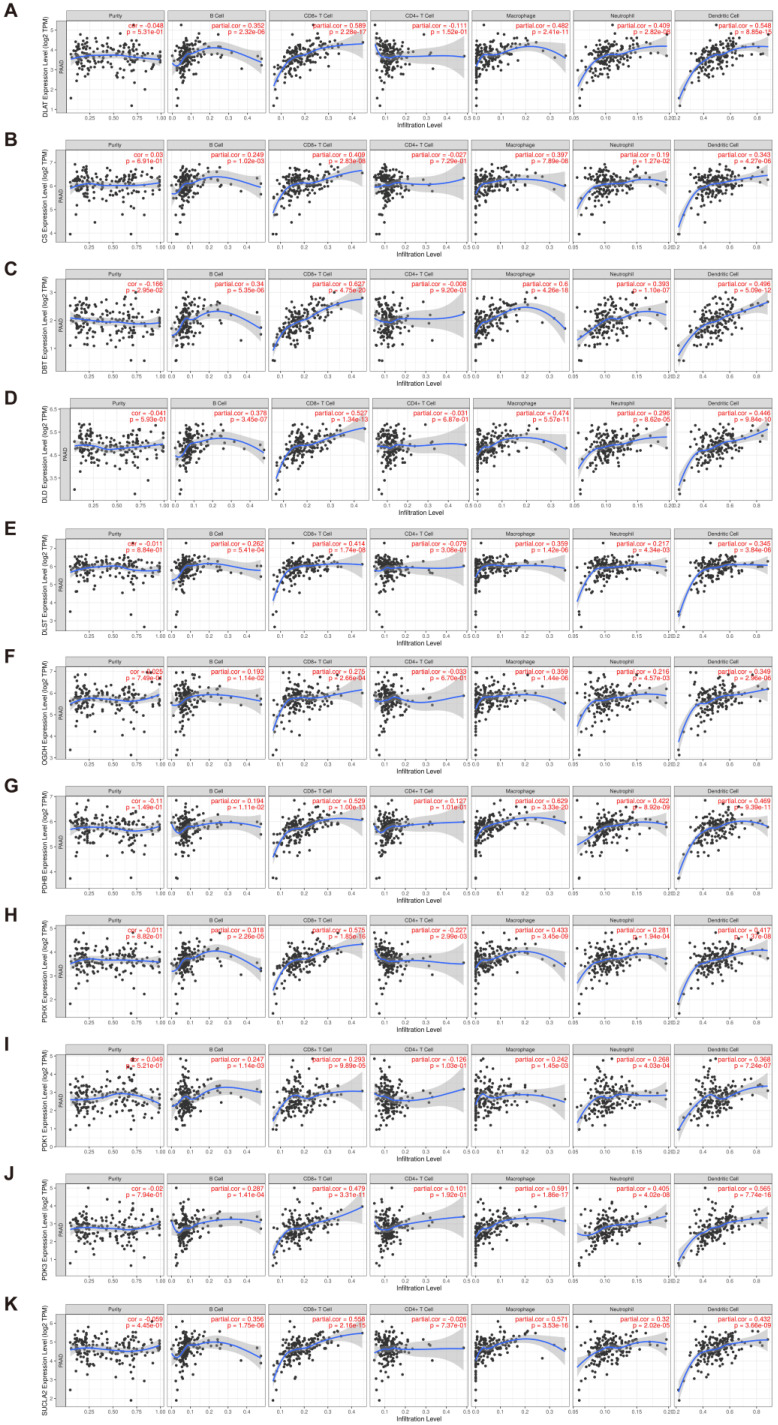
Evaluation of the infiltration abundance of TIICs, including B cells, CD8+ T cells, CD4+ T cells, macrophages, neutrophils, and dendritic cells, based on the TIMER database. (**A**) *DLAT*; (**B**) *CS*; (**C**) *DBT*; (**D**) *DLD*; (**E**) *DLST*; (**F**) *OGDH*; (**G**) *PDHB*; (**H**) *PDHX*; (**I**) *PDK1*; (**J**) *PDK3*; (**K**) *SUCLA2*.

In performing the functional enrichment analysis, we noted that many immune response-related pathways were significantly enriched. Therefore, it is reasonable to speculate that *DLAT* may be related to immune infiltration in the tumor microenvironment. The analysis results on the TIMER database showed that *DLAT* was significantly correlated with the infiltration abundance of B cells (cor = 0.352), CD8+ T cells (cor = 0.589), macrophages (cor = 0.482), neutrophils (cor = 0.409), and dendritic cells (cor = 0.548) in PAAD (Figure 10A). Additionally, *DLAT* may also regulate immune infiltration by affecting molecules with which it interacts or colocalizes. Genes that overlapped in networks previously constructed via the STRING database and the GeneMANIA database were defined as key genes, which included *CS*, *DBT*, *DLD*, *OGDH*, *PDHA1*, *PDHB*, *PDHX*, *PDK1*, *PDK3*, *SUCLA2*, and *SUCLG1*. By employing the TIMER database, we found that most of the key genes (except *PDHA1* and *SUCLG1*) were significantly correlated with TIIC infiltration (Figure 10B–K). Among them, *DBT*, *DLD*, *PDHB*, *PDHX*, *PDK3*, and *SUCLA2* were strongly correlated (|r| > 0.4, *p* < 0.05) with the infiltration level of multiple TIIC types.

**Figure 11:**
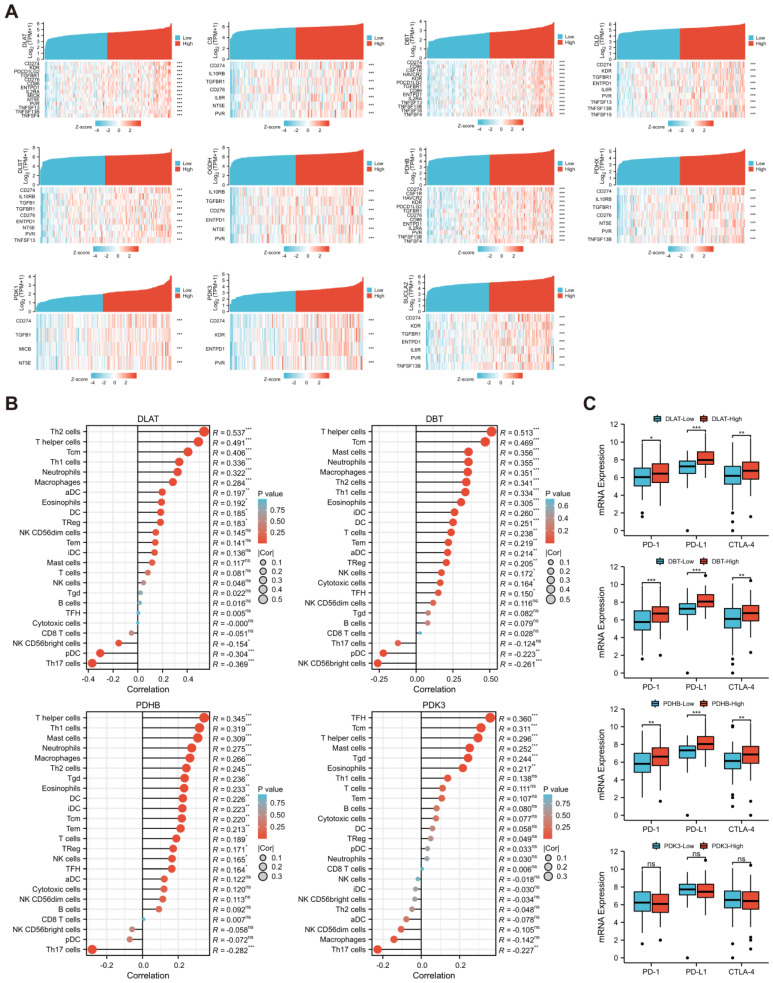
Comprehensive analysis of the effect of *DLAT* and related key genes on immune infiltration. (**A**) The correlations between *DLAT* and related key genes and the expression of immunomodulatory genes; (**B**) the results of ssGSEA revealed the influence of *DLAT* and related key genes on the infiltration abundance of 24 types of TIICs; (**C**) the differences in PD-1, PD-L1, and CTLA-4 expression between TCGA-PAAD samples grouped according to the expression of key immune-related genes (ns: no significance; * *p* value < 0.05; ** *p* value < 0.01; *** *p* value < 0.001).

**Figure 12:**
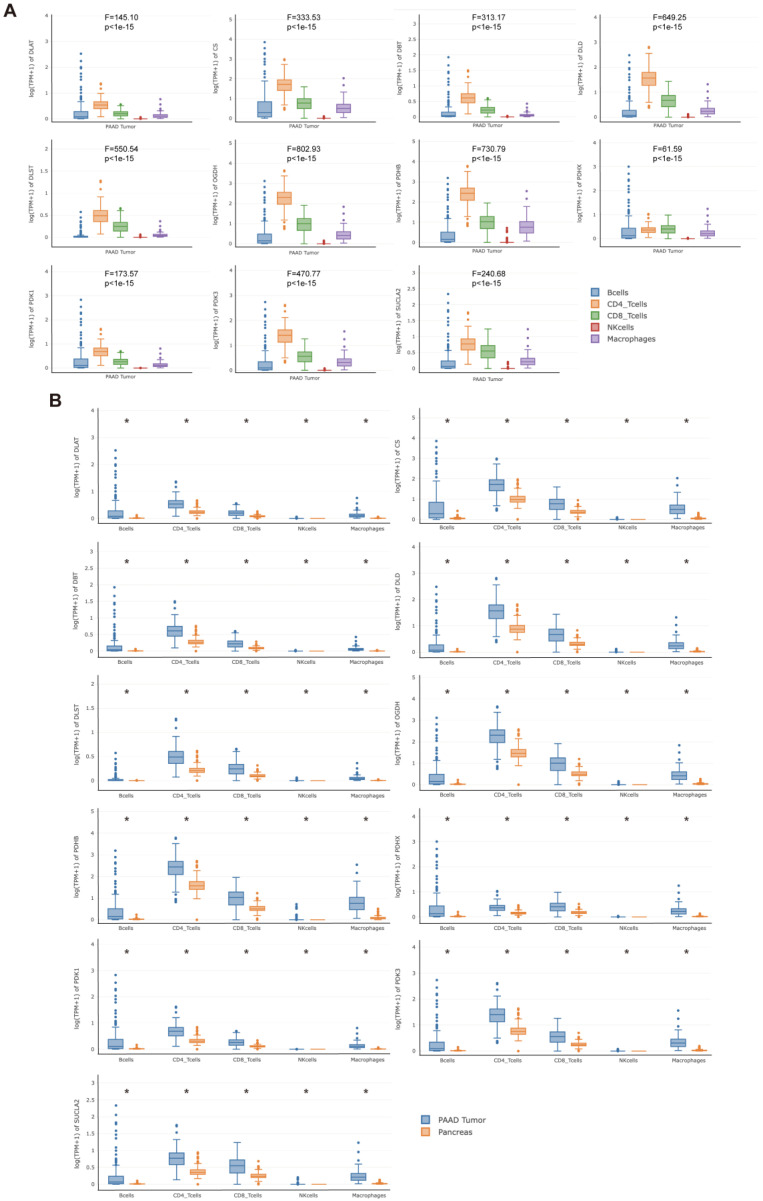
The expression of *DLAT* and related key genes in TIICs was analyzed based on the GEPIA2021 database. (**A**) Differences in the expression of *DLAT* and related key genes among different TIICs in the PAAD microenvironment; (**B**) differences in the expression of *DLAT* and related key genes between the same type of immune cells infiltrating tumor or normal tissue (* *p* value < 0.05).

Subsequently, the correlations between *DLAT* and related key genes and the expression of immunomodulatory genes were analyzed (Figure 11A). Among them, *DLAT*, *DBT*, and *PDHB* were strongly correlated (|r| > 0.4, *p* < 0.05) with more immunomodulatory genes, particularly immunoinhibitors. Based on the ESTIMATE algorithm, we found that *DLAT*, *DBT*, *PDHB*, and *PDK3* have significant effects on the ES in PAAD samples (Figure S1). Based on the above results, we defined *DLAT*, *DBT*, *PDHB*, and *PDK3* as key immune-related genes. Using ssGSEA and the CIBERSORT algorithm, we verified the significant correlation between the key immune-related genes and the infiltration levels of TIICs (Figure 11B and Figure S2). In addition, there were significant differences in the expression of PD-1, PD-L1, and CTLA-4 among samples with high or low expression of *DLAT*, *DBT*, and *PDHB*, while there were no differences in samples grouped according to *PDK3* expression level (Figure 11C). Finally, the GEPIA2021 database was used to analyze the expression of *DLAT* and related key genes in TIICs. The expression of these genes was significantly different among different TIICs in the PAAD microenvironment (Figure 12A), and also significantly different for the same type of immune cells infiltrating tumor or normal tissue (Figure 12B).

## 4. Discussion

In the present study, based on the research performed by Tsvetkov et al. [12], we defined 13 genes as CRGs, namely, *FDX1*, *LIPT1*, *LIAS*, *DLD*, *DLAT*, *PDHA1*, *PDHB*, *DBT*, *GCSH*, *DLST*, *ATP7A*, *ATP7B*, and *SLC31A1*. Among them, *FDX1*, *LIPT1*, *LIAS*, *DLD*, *DLAT*, *PDHA1*, and *PDHB* have been demonstrated to play significant roles in cuproptosis by genome-wide CRISPR–Cas9 loss-of-function screens [12]; *DLAT*, *DBT*, *GCSH*, and *DLST* are coding genes for the only four enzymes on which lipoylation occurs [29,30]; *ATP7A*, *ATP7B*, and *SLC31A1* are the genes encoding copper transporters located on the cell membrane [31]. By exploring the differential expression of CRGs between tumor and normal tissues and performing survival analyses, we identified *DLAT* as the only prognostic CRG in PAAD. To the best of our knowledge, there have been no previous studies examining the correlation between *DLAT* and PAAD progression.

Dihydrolipoamide S-acetyltransferase, the protein encoded by *DLAT*, functions as a subunit of the pyruvate dehydrogenase (PDH) complex [32]. The PDH complex irreversibly decarboxylates pyruvate to acetyl coenzyme A, thereby linking glycolysis to the TCA cycle [33]. PDH complex activity is mainly regulated by pyruvate dehydrogenase kinase (PDK), the dysregulation of which correlates with the pathobiology of many disorders of metabolic integration, including cancer [34,35]. Inhibition of the PDH complex induces a “glycolytic transition”, whereby affected cells favor the production of ATP through glycolysis, which coincides with the Warburg effect in cancers [36]. The results of the functional enrichment analysis in our study similarly suggest that the canonical functions of *DLAT* are closely related to metabolism, particularly carbon metabolism. Nevertheless, with the discovery of the novel form of cell death, cuproptosis, *DLAT* has been found to have a noncanonical function in mediating cuproptosis by binding to copper based on the lipoylated property.

To further investigate the potential biological function of *DLAT* in PAAD, we performed GSEA based on the RNA-seq data of TCGA-PAAD samples. The results showed that multiple tumor-promoting, inflammatory-related, and immune-related signaling pathways were significantly enriched in *DLAT*-high samples. Numerous highly enriched signaling pathways, such as the PI3K/Akt and MAPK pathways, have been proven to be closely related to metabolism by various studies [37,38]. This evidence revealed that the effect of *DLAT* on PAAD progression may depend on its metabolic functions. However, as the role of cuproptosis in tumor progression remains unclear, whether *DLAT* can also contribute to PAAD progression through its noncanonical function (mediating cuproptosis) needs to be further studied.

In theory, the immune system can recognize and eliminate cancerous cells to suppress cancer initiation [39]. However, as the tumor progresses, cancer cells weaken or even block the immune system’s ability to suppress the tumor based on a variety of mechanisms, which is the troublesome phenomenon called immunoescape [40]. As mentioned above, the GSEA results showed that many immune response-related biological processes or signaling pathways were enriched, which aroused our strong interest. Therefore, we performed an immune infiltration evaluation in the present study. By comprehensive analysis, we identified significant correlations between *DLAT* and immune infiltration in PAAD. The expression of *DLAT* and the related genes *DBT*, *PDHB*, and *PDK3* was significantly correlated with the infiltration abundance of TIICs in the PAAD microenvironment and the expression levels of multiple immunomodulators, particularly immunoinhibitors. Further analysis showed that the infiltrating abundance of Th cells, especially Th2 cells, had the greatest correlation with *DLAT* expression in PAAD. Th1/Th2 cells play corresponding roles in regulating immune response by secreting Th1 or Th2 cytokines, respectively. Th1 cells are essential for inducing and maintaining antitumor toxic T lymphocytes (CTLs) responses, while Th2 cells may destroy Th1 cell-mediated immunity and provide a microenvironment conducive to cancer progression [41]. As Th2 cytokines, IL4 and IL13 have been reported to restrain the immune response to tumor cells and promote the proliferation of KRAS-mutant cells [42]. IL4 derived from basophils [43], and cancer-associated fibroblast-secreted thymic stromal lymphopoietin [44] induce tumor infiltration of Th2 cells and promote Th2 inflammation, which is related to the poorer survival in patients with PAAD. The correlation between *DLAT* expression and Th2 cells infiltration in PAAD further verifies the protumorigenic effect and prognostic value of *DLAT*, and reflects the potential relationship between *DLAT* and antitumor immunity. Extensive studies have shown that overactivation of immune checkpoint-related signaling pathways in tumors plays a vital role in immunoescape [45]. Hence, we further investigated the effect of *DLAT*, *DBT*, *PDHB*, and *PDK3* on the expression of PD-1, PD-L1, and CTLA-4, the targets of commonly used immune checkpoint inhibitors. The results showed that *DLAT*, *DBT*, and *PDHB* significantly affected the expression levels of PD-1, PD-L1, and CTLA-4, which revealed the value of *DLAT*, *DBT*, and *PDHB* as biomarkers for evaluating the sensitivity of immunotherapy. The emergence of immune checkpoint blockade therapy has greatly changed the strategy of cancer treatment, but some patients do not benefit from this treatment [46]. In-depth studies of the mechanisms by which *DLAT*, *DBT*, and *PDHB* influence immune infiltration might contribute to the development of new combination therapies that enhance the efficacy of immunotherapy.

Based on drug sensitivity data from the CTRP and the GDSC, we investigated the association of *DLAT* with therapy resistance. We found that PAAD patients with high *DLAT* expression exhibited resistance to multiple drugs. The correlation between *DLAT* and inhibitors of the phosphoinositide 3-kinase (PI3K)/Akt/mTOR or mitogen-activated protein kinase (MAPK) signaling pathways was the most prominent. For PAAD patients, high expression of *DLAT* significantly enhances resistance to these agents, which include AZD2014 (vistusertib), pictilisib, dactolisib, etc. This phenomenon is consistent with the results of functional enrichment analysis in which the PI3K/Akt and MAPK pathways were enriched in *DLAT*-high samples. Furthermore, *DLAT* expression was similarly significantly correlated with commonly used chemotherapeutics in PAAD, such as gemcitabine, oxaliplatin, 5-fluorouracil, and irinotecan, revealing a crucial function of *DLAT* in chemoresistance. However, whether *DLAT*, as a key molecule mediating cuproptosis, can bring new breakthroughs for cancer therapy deserves more attention. Before the mechanism of cuproptosis was elucidated, scientists had tried many different ways to use copper for cancer treatment; these candidate treatments included copper chelators, copper ionophores, copper-based nanomaterials, etc. [47,48]. The high expression of *DLAT* indicates susceptibility to cuproptosis, which may contribute to the precise application of copper-based cancer therapy. In fact, cuproptosis was discovered in conjunction with the study of cellular adaptation to proteotoxic stress [11]. Tsvetkov et al. revealed that when cells were forced to use oxidative phosphorylation rather than glycolysis, they became resistant to proteasome inhibitors and more sensitive to elesclomol, a copper ionophore, and cuproptosis is one of the mechanisms of cell death induced by elesclomol [10]. Therefore, in patients with high *DLAT* expression, the combination of proteasome inhibitors and copper ionophores may achieve better therapeutic effects.

Nevertheless, the present study has several limitations. First, it is a retrospective study based on data from open-access databases, and further prospective studies are needed to confirm the prognostic value of *DLAT* in PAAD. Second, this study was based on bioinformatics analysis with a lack of experimental evidence to elucidate the mechanisms by which *DLAT* influences tumor progression, chemoresistance, and immune infiltration. Last, the process of translating these basic scientific breakthroughs into clinical applications will be a formidable challenge.

## 5. Conclusions

In conclusion, our study preliminarily revealed the prognostic value of *DLAT*, which is correlated with PAAD progression, chemoresistance, and immune infiltration, providing a valuable reference for PAAD treatment. However, our findings need to be confirmed by further in vivo and in vitro experiments.

## Figures and Tables

**Table 1:** Univariate Cox analysis for screening prognostic CRGs (* *p* value < 0.05).

Gene	HR	95% CI	*p* Value
*ATP7A*	1.17	0.50–2.69	0.720
*DLAT*	2.72	1.10–6.74	0.030 *
*DLST*	1.58	0.64–3.87	0.318
*FDX1*	0.77	0.24–2.51	0.666
*LIPT1*	0.45	0.16–1.25	0.124
*PDHA1*	0.70	0.20–2.41	0.568
*PDHB*	1.35	0.33–5.55	0.681

## Data Availability

The datasets presented in this study can be found in online databases. The names of the databases and accession numbers can be found in the article.

## Supplementary Materials

The following supporting information can be downloaded at: https://www.mdpi.com/article/10.3390/curroncol30030228/s1, Figure S1: The effect of *DLAT* and related key genes on the SS, IS, and ES for PAAD samples. Figure S2: Immune cell infiltration analysis based on CIBERSORT algorithm. Figure S3: The whole western blotting data. Table S1: List of immunomodulatory genes acquired from the TISIDB. Table S2: Results of GSEA according to the “h.all.v7.5.symbols.gmt” gene set. Table S3: Results of GSEA according to the “c2.cp.kegg.v7.5.symbols.gmt” gene set. Table S4: Correlation between *DLAT* and the IC50 values of multiple drugs calculated based on the data from the GDSC database. Table S5: Correlation between *DLAT* and the IC50 values of multiple drugs calculated based on the data from the CTRP.
