# Supplementary material for: Cuproptosis-Related Gene DLAT as a Novel Biomarker Correlated with Prognosis, Chemoresistance, and Immune Infiltration in Pancreatic Adenocarcinoma: A Preliminary Study Based on Bioinformatics Analysis
Source: Curr Oncol. 2023 Mar 2;30(3):2997–3019. doi: 10.3390/curroncol30030228 (PMC10047569; doi:10.3390/curroncol30030228)
Supplement: Supplementary file 1 [file curroncol-30-00228-s001.zip › curroncol-2200800-supplementary.pdf]

Supplementary materials

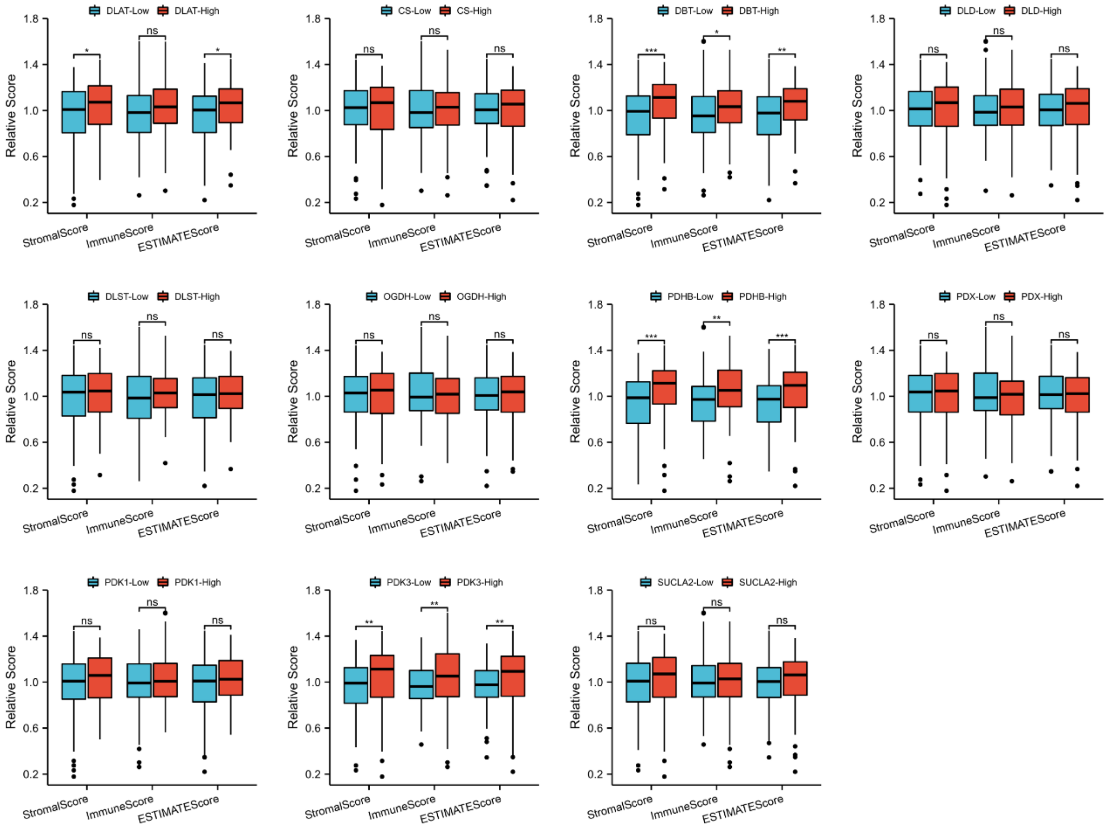

Figure S1 The effect of *DLAT* and related key genes on the SS, IS and ES for PAAD samples (ns: no significance; **p* value < 0.05; ***p* value < 0.01; ****p* value < 0.001).

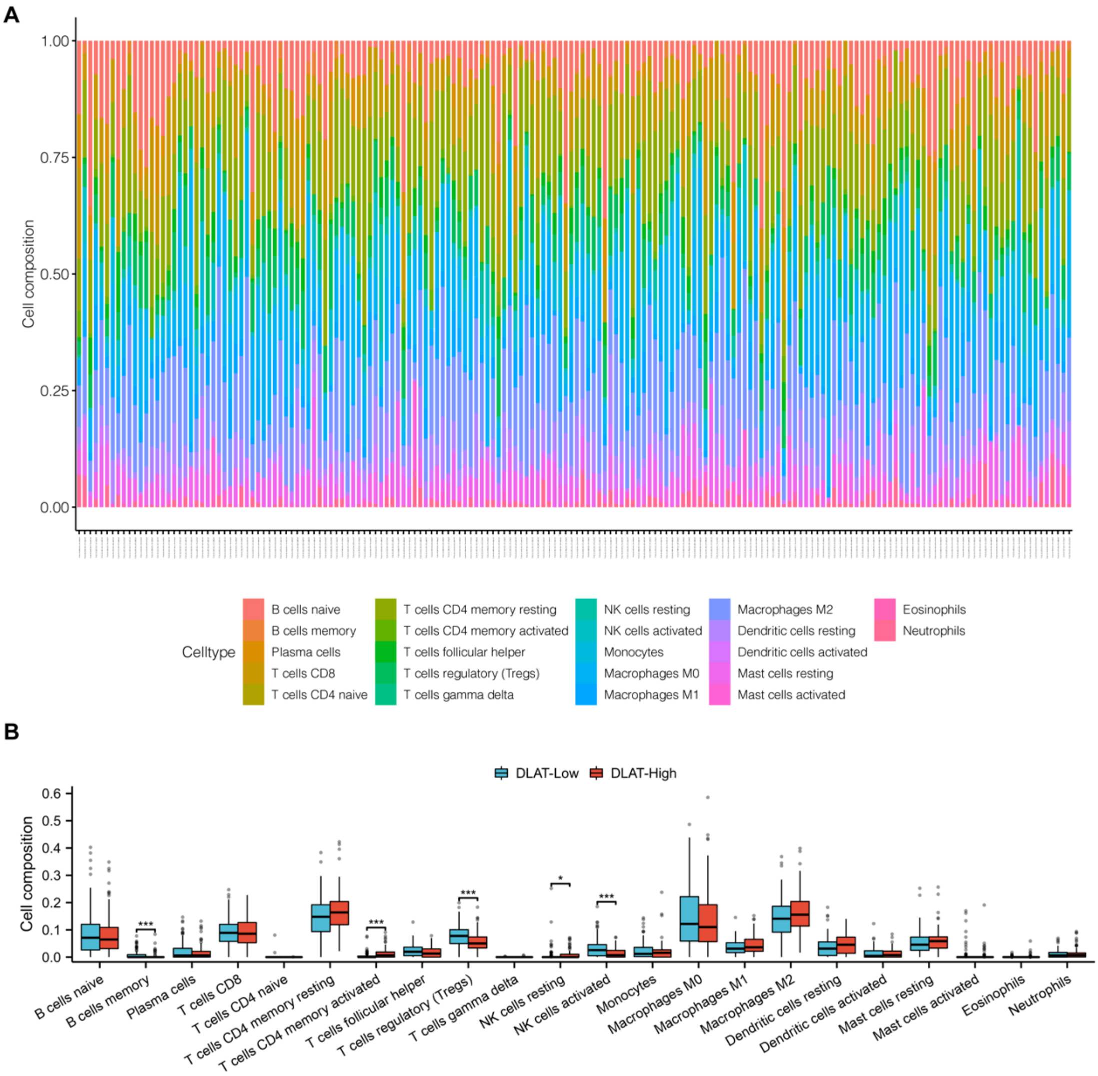

Figure S2 Immune cell infiltration analysis based on CICBERSORT algorithm. (A) The convolution histogram shows the proportion of immune cells in different samples; (B) the difference in the immune cell abundance between *DLAT*-high and *DLAT*-low PAAD samples (*p value < 0.05; **p value < 0.01; ***p value < 0.001).

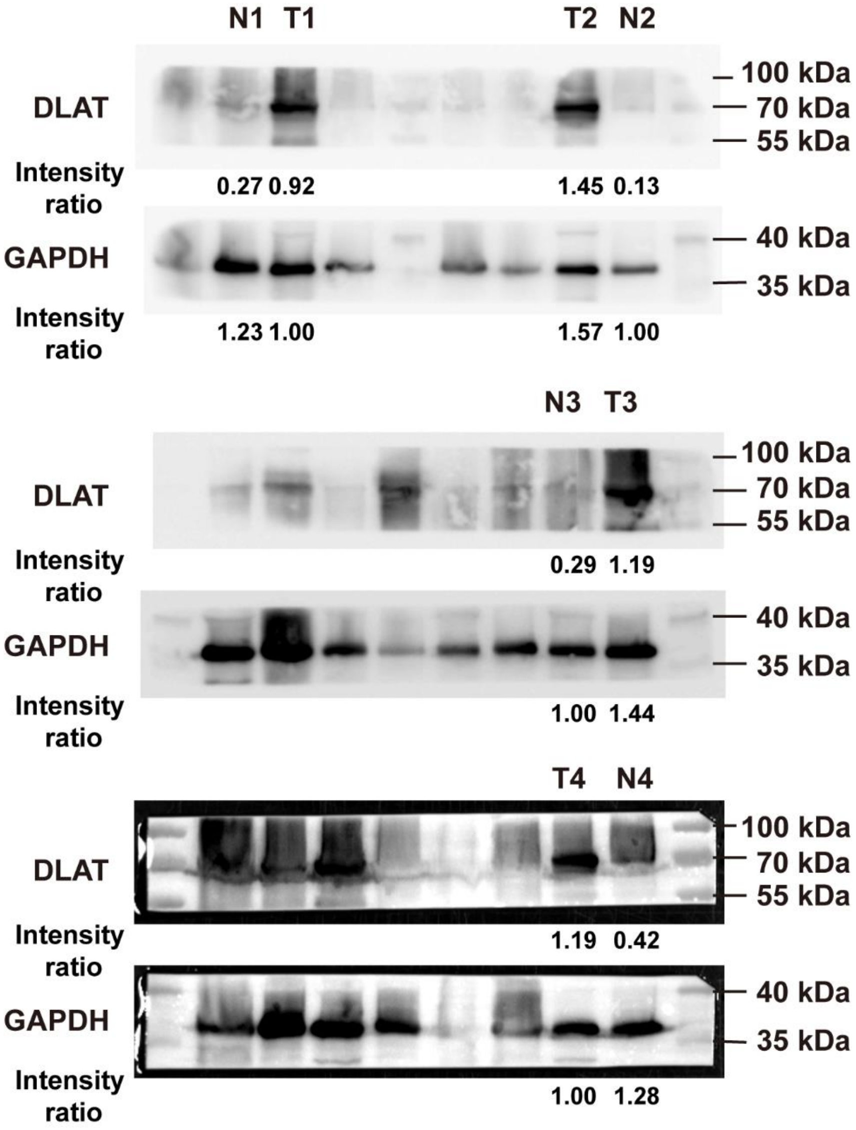

Figure S3. Western Blotting data

.

Table S1 List of immunomodulatory genes obtained from the TISIDB database.

Gene	Protein	Description
ADORA2A	A2aR	Immunoinhibitor
BTLA	CD272	Immunoinhibitor
CD160	CD160	Immunoinhibitor
CD244	SLAMF4	Immunoinhibitor
CD274	PD-L1	Immunoinhibitor
CD96	CD96	Immunoinhibitor
CSF1R	CSF1R	Immunoinhibitor
CTLA4	CTLA-4	Immunoinhibitor
HAVCR2	TIM3	Immunoinhibitor
IDO1	IDO1	Immunoinhibitor
IL10	IL10	Immunoinhibitor
IL10RB	IL10RB	Immunoinhibitor
KDR	VEGFR	Immunoinhibitor
KIR2DL1	KIR2DL1	Immunoinhibitor
KIR2DL3	KIR2DL3	Immunoinhibitor
LAG3	LAG3	Immunoinhibitor
LGALS9	LGALS9	Immunoinhibitor
PDCD1	PD-1	Immunoinhibitor
PDCD1LG2	PD-L2	Immunoinhibitor
PVRL2	CD112	Immunoinhibitor
TGFB1	TGFB1	Immunoinhibitor
TGFBR1	TGFBR1	Immunoinhibitor
TIGIT	TIGIT	Immunoinhibitor
VTCN1	B7-H4	Immunoinhibitor
BTNL2	BTNL2	Immunostimulator
C10orf54	VISTA	Immunostimulator
CD27	CD27	Immunostimulator
CD276	B7-H3	Immunostimulator
CD28	CD28	Immunostimulator
CD40	CD40	Immunostimulator
CD40LG	CD40LG	Immunostimulator
CD48	CD48	Immunostimulator
CD70	CD70	Immunostimulator
CD80	B7.1	Immunostimulator
CD86	B7.2	Immunostimulator
CXCL12	CXCL12	Immunostimulator
CXCR4	CXCR4	Immunostimulator
ENTPD1	CD39	Immunostimulator
HHLA2	B7-H7	Immunostimulator
ICOS	ICOS	Immunostimulator
ICOSLG	B7-H2	Immunostimulator
IL2RA	CD25	Immunostimulator
IL6	IL6	Immunostimulator
IL6R	IL6R	Immunostimulator
KLRC1	NKG2A	Immunostimulator
KLRK1	NKG2D	Immunostimulator
LTA	LTA	Immunostimulator
MICB	MICB	Immunostimulator
NT5E	CD73	Immunostimulator
PVR	CD155	Immunostimulator
RAET1E	RAET1E	Immunostimulator
TMEM173	STING	Immunostimulator
TMIGD2	TMIGD2	Immunostimulator
TNFRSF13B	CD267	Immunostimulator
TNFRSF13C	BAFF-R	Immunostimulator
TNFRSF14	HVEM	Immunostimulator

TNFRSF17	BCMA	Immunostimulator
TNFRSF18	GITR	Immunostimulator
TNFRSF25	DR3	Immunostimulator
TNFRSF4	OX40	Immunostimulator
TNFRSF8	CD30	Immunostimulator
TNFRSF9	4-1BB	Immunostimulator
TNFSF13	APRIL	Immunostimulator
TNFSF13B	BAFF	Immunostimulator
TNFSF14	LIGHT	Immunostimulator
TNFSF15	TL1A	Immunostimulator
TNFSF18	GITRL	Immunostimulator
TNFSF4	OX-40L	Immunostimulator
TNFSF9	4-1BB-L	Immunostimulator
ULBP1	ULBP1	Immunostimulator

Table S2 Results of GSEA according to the “h.all.v7.5.symbols.gmt” gene set.

Description	enrichmentScore	NES	p.adjust
HALLMARK_ALLOGRAFT_REJECTION	0.5013659	2.35863664	0.00527872
HALLMARK_KRAS_SIGNALING_UP	0.42207929	1.98563901	0.00527872
HALLMARK_IL2_STAT5_SIGNALING	0.31685522	1.49062061	0.00527872
HALLMARK_EPITHELIAL_MESENCHYMAL_ TRANSITION	0.57519248	2.70854383	0.00527872
HALLMARK_INFLAMMATORY_RESPONSE	0.54597787	2.56661821	0.00527872
HALLMARK_G2M_CHECKPOINT	0.46771582	2.19871171	0.00527872
HALLMARK_INTERFERON_GAMMA_RESPONSE	0.43029524	2.02623217	0.00527872
HALLMARK_MITOTIC_SPINDLE	0.37611089	1.76808094	0.00527872
HALLMARK_COMPLEMENT	0.36892312	1.73723488	0.00527872
HALLMARK_TNFA_SIGNALING_VIA_NFKB	0.36604056	1.72366111	0.00527872
HALLMARK_E2F_TARGETS	0.36450068	1.71640988	0.00527872
HALLMARK_APICAL_JUNCTION	0.32721236	1.53821107	0.00527872
HALLMARK_MTORC1_SIGNALING	0.313446	1.47349601	0.00527872
HALLMARK_UV_RESPONSE_DN	0.48446772	2.17806131	0.00527872
HALLMARK_PROTEIN_SECRETION	0.39041863	1.67295604	0.00527872
HALLMARK_IL6_JAK_STAT3_SIGNALING	0.49021913	2.06828973	0.00527872
HALLMARK_OXIDATIVE_PHOSPHORYLATION	-0.3182138	-1.5682477	0.00847601
HALLMARK_ANDROGEN_RESPONSE	0.39301266	1.70296723	0.01350621
HALLMARK_DNA_REPAIR	-0.3233591	-1.5387949	0.02837282
HALLMARK_ANGIOGENESIS	0.44300819	1.54835578	0.07220217
HALLMARK_APOPTOSIS	0.2956582	1.34362362	0.07643507
HALLMARK_KRAS_SIGNALING_DN	0.27466243	1.29118866	0.09007761
HALLMARK_TGF_BETA_SIGNALING	0.37481495	1.43976282	0.09348291
HALLMARK_SPERMATOGENESIS	0.2963382	1.31022908	0.09348291
HALLMARK_INTERFERON_ALPHA_RESPONSE	0.28376059	1.22161478	0.2134647
HALLMARK_COAGULATION	-0.2498355	-1.1650413	0.27771956

Table S3 Results of GSEA according to the “c2.cp.kegg.v7.5.symbols.gmt” gene set.

Description	EnrichmentScore	NES	p.adjust
KEGG_PATHWAYS_IN_CANCER	0.36064159	1.78092983	0.03166559
KEGG_NEUROACTIVE_LIGAND_RECEPTOR_ INTERACTION	0.3607433	1.73117775	0.03166559
KEGG_CYTOKINE_CYTOKINE_RECEPTOR_ INTERACTION	0.52001746	2.47329168	0.03166559
KEGG_REGULATION_OF_ACTIN_CYTOSKELETON	0.37417044	1.75460317	0.03166559
KEGG_JAK_STAT_SIGNALING_PATHWAY	0.42375027	1.87728561	0.03166559
KEGG_CALCIUM_SIGNALING_PATHWAY	0.34018146	1.55696811	0.03166559
KEGG_FOCAL_ADHESION	0.42281874	1.96163683	0.03166559
KEGG_CHEMOKINE_SIGNALING_PATHWAY	0.4586855	2.10660463	0.03166559
KEGG_CELL_ADHESION_MOLECULES_CAMS	0.52200133	2.29439776	0.03166559
KEGG_NATURAL_KILLER_CELL_MEDIATED_ CYTOTOXICITY	0.40046709	1.72936801	0.03166559
KEGG_SMALL_CELL_LUNG_CANCER	0.47804896	1.96698177	0.03166559
KEGG_ECM_RECEPTOR_INTERACTION	0.58118667	2.38493276	0.03166559
KEGG_T_CELL_RECEPTOR_SIGNALING_PATHWAY	0.40729875	1.72851959	0.03166559
KEGG_SYSTEMIC_LUPUS_ERYTHEMATOSUS	0.44086483	1.883665	0.03166559
KEGG_HEMATOPOIETIC_CELL_LINEAGE	0.57546919	2.35590459	0.03166559
KEGG_ADHERENS_JUNCTION	0.44666634	1.79019868	0.03166559
KEGG_VIRAL_MYOCARDITIS	0.43680132	1.73082973	0.03166559
KEGG_LEISHMANIA_INFECTION	0.54807368	2.17324032	0.03166559
KEGG_ANTIGEN_PROCESSING_AND_ PRESENTATION	0.44736538	1.76767653	0.03166559
KEGG_INTESTINAL_IMMUNE_NETWORK_FOR_ IGA_PRODUCTION	0.63642265	2.32535782	0.03166559
KEGG_TYPE_I_DIABETES_MELLITUS	0.57607759	2.05755354	0.03166559
KEGG_PRIMARY_IMMUNODEFICIENCY	0.55509686	1.90882835	0.03166559
KEGG_ALLOGRAFT_REJECTION	0.57359304	1.98948177	0.03166559
KEGG_ASTHMA	0.5879972	1.8787335	0.03166559
KEGG_GRAFT_VERSUS_HOST_DISEASE	0.60988361	2.13039835	0.03166559
KEGG_RIBOSOME	-0.5392374	-2.307014	0.03723555
KEGG_PARKINSONS_DISEASE	-0.4235233	-1.8956174	0.03723555
KEGG_OXIDATIVE_PHOSPHORYLATION	-0.4942051	-2.2215101	0.03723555
KEGG_HUNTINGTONS_DISEASE	-0.3324662	-1.5930316	0.03784286
KEGG_AXON_GUIDANCE	0.37344459	1.64050805	0.04191213
KEGG_ARRHYTHMOGENIC_RIGHT_VENTRICULAR_ CARDIOMYOPATHY_ARVC	0.44474556	1.78902637	0.04191213
KEGG_NOD_LIKE_RECEPTOR_SIGNALING_ PATHWAY	0.4476943	1.74611576	0.04191213
KEGG_CELL_CYCLE	0.35641065	1.56005346	0.05373718
KEGG_LEUKOCYTE_TRANSENDOTHELIAL_ MIGRATION	0.38568719	1.64790974	0.05476678
KEGG_AUTOIMMUNE_THYROID_DISEASE	0.50738156	1.78419533	0.0550724
KEGG_APOPTOSIS	0.41447295	1.70539173	0.06384647
KEGG_HYPERTROPHIC_CARDIOMYOPATHY_HCM	0.39645994	1.62689607	0.06384647
KEGG_B_CELL_RECEPTOR_SIGNALING_PATHWAY	0.41288708	1.66087294	0.07154866
KEGG_TOLL_LIKE_RECEPTOR_SIGNALING_PATHWAY	0.40792787	1.68994975	0.07154866
KEGG_DILATED_CARDIOMYOPATHY	0.40073203	1.66013906	0.07154866
KEGG_O_GLYCAN_BIOSYNTHESIS	0.51671279	1.68839978	0.07401048
KEGG_PROSTATE_CANCER	0.3806964	1.57713612	0.07913119
KEGG_MELANOMA	0.42157162	1.67048189	0.07981617
KEGG_PANCREATIC_CANCER	0.40061382	1.59507942	0.0854382
KEGG_OOCYTE_MEIOSIS	0.34265473	1.45806988	0.10767099
KEGG_GLYCINE_SERINE_AND_THREONINE_ METABOLISM	-0.4695024	-1.6250524	0.1143012
KEGG_PROGESTERONE_MEDIATED_OOCYTE_ MATURATION	0.36964425	1.51685633	0.1351811
KEGG_FC_GAMMA_R_MEDIATED_PHAGOCYTOSIS	0.35382878	1.47320335	0.14052354
KEGG_VASCULAR_SMOOTH_MUSCLE_ CONTRACTION	0.3341269	1.42761023	0.14052354
KEGG_LONG_TERM_DEPRESSION	0.370469	1.4689962	0.14783
KEGG_TGF_BETA_SIGNALING_PATHWAY	0.36131908	1.48917345	0.15564561
KEGG_PROXIMAL_TUBULE_BICARBONATE_ RECLAMATION	-0.4883714	-1.540467	0.16461307
KEGG_COLORECTAL_CANCER	0.37987228	1.48159353	0.16665924

KEGG_MAPK_SIGNALING_PATHWAY	0.26443739	1.27225698	0.18753899
KEGG_ENDOMETRIAL_CANCER	0.38281489	1.44963182	0.20968633
KEGG_FC_EPSILON_RI_SIGNALING_PATHWAY	0.34479496	1.3916741	0.21034821
KEGG_GAP_JUNCTION	0.33237138	1.3711064	0.21523601
KEGG_RENAL_CELL_CARCINOMA	0.346182	1.37835432	0.21523601
KEGG_NON_SMALL_CELL_LUNG_CANCER	0.37294504	1.41984613	0.21780918
KEGG_STARCH_AND_SUCROSE_METABOLISM	0.37674891	1.39833548	0.22953931
KEGG_GLIOMA	0.34773844	1.37447884	0.2323431
KEGG_RENIN_ANGIOTENSIN_SYSTEM	0.50791321	1.45350425	0.25022365
KEGG_ALZHEIMERS_DISEASE	-0.2603412	-1.2329537	0.26422759
KEGG_COMPLEMENT_AND_COAGULATION_ CASCADES	-0.3270652	-1.3383664	0.26616503
KEGG_ETHER_LIPID_METABOLISM	0.40361484	1.36170339	0.27776662
KEGG_WNT_SIGNALING_PATHWAY	0.27634468	1.23763491	0.28637824
KEGG_CHRONIC_MYELOID_LEUKEMIA	0.32282178	1.29384075	0.29115217
KEGG_TYROSINE_METABOLISM	-0.3534909	-1.3086019	0.29678693
KEGG_PENTOSE_AND_GLUCURONATE_ INTERCONVERSIONS	0.42931473	1.37172075	0.29678693
KEGG_STEROID_HORMONE_BIOSYNTHESIS	0.35647224	1.34987827	0.29678693
KEGG_NEUROTROPHIN_SIGNALING_PATHWAY	0.28687733	1.25703454	0.29678693

Table S4 Correlation between DLAT and the IC50 values of multiple drugs calculated based on the data from the GDSC database.

Drug	Correlation	p value
ERK Inhibitor	0.4906716	5.69E-10
Dactinomycin	0.45385104	1.41E-08
AZD2014	0.44097483	3.96E-08
Palbociclib	0.39868297	8.92E-07
VSP34	0.39656159	1.03E-06
Pictilisib	0.38536023	2.18E-06
Irinotecan	0.38191403	2.73E-06
Elephantin	0.36541508	7.76E-06
Topotecan	0.35558278	1.41E-05
Dactolisib	0.35059937	1.89E-05
Camptothecin	0.34087712	3.31E-05
Mirin	0.3407109	3.34E-05
VX-11e	0.33568514	4.43E-05
Oxaliplatin	0.33426335	4.79E-05
GNE-317	0.33232304	5.33E-05
Dabrafenib	0.33209804	5.40E-05
Gemcitabine	0.33013004	6.01E-05
Teniposide	0.31159696	0.0001602
Uprosertib	0.30967093	0.00017673
AZD5153	0.30822632	0.00019015
Epirubicin	0.29834477	0.00031065
5-Fluorouracil	0.29457778	0.00037288
OSI-027	0.28781015	0.00051443
PF-4708671	0.28442486	0.00060249
Cytarabine	0.27662752	0.00086058
Mitoxantrone	0.26372003	0.0015184
Nutlin-3a (-)	0.25742006	0.00198349
Sabutoclax	0.25694215	0.00202357
Vinorelbine	0.25690328	0.00202686
Leflunomide	0.25474286	0.00221767
Eg5	0.25459971	0.00223087
Rapamycin	0.24558245	0.00322082
ULK1 Inhibitor	0.24557837	0.00322134
Pevonedistat	0.24211937	0.003696
Cisplatin	0.23349376	0.00516481
Fulvestrant	0.23304717	0.00525342
PRIMA-1MET	0.23295226	0.00527242
Foretinib	0.23097635	0.0056823
PD0325901	0.22984704	0.00592912
SB505124	0.22917314	0.00608092
Lapatinib	0.22308678	0.00761665
Niraparib	0.22128695	0.00813236
Vorinostat	0.20790826	0.01303514
Afuresertib	0.20275601	0.01552254
Obatoclax Mesylate	0.20055537	0.016705
OTX015	0.19572824	0.01957574
Talazoparib	0.19522327	0.01989934
Selumetinib	0.18750176	0.02545356
Buparlisib	0.18579854	0.02684331
Dihydrorotenone	0.18416353	0.02823786
Docetaxel	0.18146988	0.03066998
GSK269962A	0.18021332	0.03186407
JAK1 Inhibitor	0.17880905	0.03324494
Wnt-C59	0.1759958	0.0361641
Sorafenib	0.17506761	0.03717347
MK-2206	0.1750612	0.03718052

Trametinib	0.17065605	0.04230089
WZ4003	0.15113649	0.0725886
AZD8055	0.15087061	0.07309877
AZD1332	0.14933623	0.07609986
AT13148	0.14744171	0.07994169
LGK974	0.13756081	0.10257247
SCH772984	0.13058238	0.12139249
XAV939	0.13034655	0.1220723
Pyridostatin	0.12317036	0.14419722
PRT062607	0.12058107	0.15288638
AZD5438	0.12032376	0.15377094
CDK9 Inhibitor	0.11561371	0.17064994
AZD7762	0.11510115	0.17256651
Ulixertinib	0.11471887	0.17400627
Ipatasertib	0.11380228	0.17749448
Podophyllotoxin bromide	0.11352089	0.17857565
IGF1R Inhibitor	0.1125811	0.18222153
Axitinib	0.11174047	0.18552879
VE-822	0.10968352	0.19380607
Tamoxifen	0.10778675	0.20167357
PLX-4720	0.10382571	0.21884062
Alisertib	0.10203641	0.22692664
Entinostat	0.10168281	0.22854921
GSK2578215A	0.0975302	0.2482177
IRAK4	0.09635965	0.25396754
KRAS (G12C) Inhibitor-12	0.09568768	0.2573094
Sapitinib	0.09558156	0.25783994
AZD5363	0.09239621	0.27411399
Telomerase Inhibitor IX	0.09140267	0.27932902
Olaparib	0.091192	0.28044331
AZD6738	0.09049998	0.28412472
AZ6102	0.09017872	0.2858447
GDC0810	0.08950062	0.28949789
Temozolomide	0.08773998	0.29912798
MIRA-1	0.08769688	0.29936635
GSK1904529A	0.07918688	0.34888602
PAK	0.07792226	0.35666096
TAF1	0.07581206	0.36987292
I-BET-762	0.07302129	0.38780117
ML323	0.07151339	0.39770241
AZD4547	0.06921739	0.41306466
MK-8776	0.06780906	0.42265729
Taselisib	0.06292176	0.45693113
AZD5582	0.06095003	0.4711839
Vinblastine	0.05865144	0.48810105
Luminespib	0.05557882	0.51121163
Ribociclib	0.05339633	0.52796387
Nilotinib	0.0529537	0.53139486
Vincristine	0.05116291	0.54538902
Alpelisib	0.0506443	0.54947524
EPZ5676	0.03583732	0.67199439
AGI-6780	0.03581469	0.67218955
P22077	0.0341756	0.68638355
Entospletinib	0.0334029	0.69311342
GSK591	0.03307862	0.69594495
I-BRD9	0.03227173	0.70300862
AGI-5198	0.03161823	0.70874812
Afatinib	0.0314582	0.71015613
VE821	0.03075177	0.71638327
Sinularin	0.03015599	0.72164967
YK-4-279	0.02930688	0.7291778
EPZ004777	0.02567457	0.76166608

LJI308	0.02295098	0.78630574
IAP	0.02219292	0.79320271
Paclitaxel	0.02145346	0.79994595
PCI-34051	0.01834336	0.82846355
Venetoclax	0.01633334	0.84701636
BDP-00009066	0.01240186	0.8835388
JAK Inhibitor	0.01189309	0.88828486
BIBR-1532	0.01060407	0.90032667
MIM1	0.00894588	0.91585026
Savolitinib	0.00866344	0.91849774
Linsitinib	0.00735402	0.93078289
Crizotinib	-0.0011554	0.98911246
OF-1	-0.0018996	0.98210033
Gefitinib	-0.0086052	0.9190433
BMS-345541	-0.0096896	0.90888341
MK-1775	-0.0112557	0.89423629
Ibrutinib	-0.0156683	0.85317399
AZ960	-0.017129	0.83966176
LCL161	-0.019345	0.81925256
NVP-ADW742	-0.0197354	0.8156695
Navitoclax	-0.0233889	0.78232865
Acetalax	-0.0244668	0.77256507
Sepantronium bromide	-0.0262691	0.75631787
AMG-319	-0.0285215	0.73616392
GSK2606414	-0.0291803	0.73030261
GSK343	-0.0297883	0.72490653
Cyclophosphamide	-0.0340703	0.68729938
Fludarabine	-0.0351214	0.67817951
Picolinici-acid	-0.0364513	0.66670778
BPD-00008900	-0.048013	0.57043465
IWP-2	-0.0480312	0.57028843
CZC24832	-0.0486573	0.56526757
Erlotinib	-0.050093	0.55383511
BMS-754807	-0.0534078	0.52787509
WIKI4	-0.0587279	0.48753304
Daporinad	-0.0614572	0.46749466
Dinaciclib	-0.0651056	0.44142865
AZD6482	-0.0652314	0.44054517
AZD8186	-0.0703451	0.40547658
Wee1 Inhibitor	-0.0709598	0.40137521
AZD1208	-0.0730836	0.38739505
Dasatinib	-0.0786043	0.35245457
MG-132	-0.0837635	0.32164734
Doramapimod	-0.0854517	0.31195665
PD173074	-0.0903256	0.28505767
Gallibiscoquinazole	-0.0928262	0.27187771
Zoledronate	-0.109892	0.19295499
PFI3	-0.1105567	0.19026038
RVX-208	-0.1123103	0.18328201
Staurosporine	-0.1222459	0.14725559
Tozasertib	-0.1284787	0.12756105
JQ1	-0.13581	0.10706338
Carmustine	-0.1445799	0.08603912
MN-64	-0.1483961	0.07798725
ABT737	-0.1523896	0.07022292
Nelarabine	-0.1654628	0.04908234
Ruxolitinib	-0.1691862	0.04413562
AZD5991	-0.1752587	0.03696378
SB216763	-0.1762146	0.03592959
LY2109761	-0.1765326	0.035591
BMS-536924	-0.179992	0.03207838
WEHI-539	-0.1920926	0.02201048

NU7441	-0.2017179	0.01607095
KU-55933	-0.2106485	0.01186008
ZM447439	-0.2222386	0.00785594
Bortezomib	-0.236488	0.00460444
AZD3759	-0.2371116	0.00449483
BI-2536	-0.2666588	0.0013375
RO-3306	-0.2782542	0.00079957
Osimertinib	-0.2869597	0.00053536
Cediranib	-0.2889436	0.00048771
UMI-77	-0.4161903	2.59E-07

Table S5 Correlation between DLAT and the IC50 values of multiple drugs calculated based on the data from the CTRP.

Drug	Correlation	p value
BRD-K79669418	0.5600572	4.28E-13
brivanib	0.53757782	5.27E-12
BRD-K16130065	0.52810897	1.44E-11
BRD-A02303741:carboplatin (1:1 mol/mol)	0.4742904	2.48E-09
BRD-K58730230	0.47030837	3.51E-09
SJ-172550	0.4532902	1.47E-08
linifanib	0.44312575	3.34E-08
BRD-K64610608	0.43552462	6.06E-08
CAY10576	0.43373633	6.96E-08
erismodegib	0.41888321	2.12E-07
selumetinib:piperlongumine (8:1 mol/mol)	0.40938436	4.22E-07
STF-31	0.40666908	5.11E-07
KH-CB19	0.39687563	1.01E-06
pyrazolanthrone	0.39317968	1.30E-06
ouabain	0.38871933	1.75E-06
linsitinib	0.38595687	2.10E-06
azacitidine	0.38168661	2.78E-06
sorafenib	0.37045226	5.68E-06
etomoxir	0.35311768	1.63E-05
FQI-1	0.34306768	2.92E-05
pitstop2	0.34247828	3.02E-05
pevonedistat	0.33367099	4.95E-05
dapori0d	0.32581849	7.60E-05
RITA	0.32294054	8.86E-05
MLN2238	0.32108215	9.78E-05
BMS-345541	0.31828513	0.00011331
skepinone-L	0.31054684	0.00016902
spautin-1	0.30955958	0.00017773
betulinic acid	0.30816449	0.00019075
CAY10618	0.30684535	0.00020387
methotrexate	0.30194752	0.00026027
NSC19630	0.2858541	0.00056375
MI-2	0.26608159	0.0013714
hyperforin	0.26541972	0.00141123
VER-155008	0.26252634	0.00159805
GMX-1778	0.26246581	0.00160219
fluorouracil	0.25968958	0.00180281
procarbazine	0.25918264	0.00184182
bexarotene	0.25502411	0.00219194
AZD7545	0.2543869	0.00225063
necrosulfo0mide	0.25380433	0.00230552
bendamustine	0.25155401	0.00252915
Platin	0.24899174	0.00280754
PHA-793887	0.24182669	0.00373891
TW-37	0.24131068	0.00381567
ML029	0.23768053	0.00439687
CIL70	0.23742417	0.00444077
BRD-K97651142	0.23204065	0.00545812
gemcitabine	0.23158829	0.00555242
CR-1-31B	0.23013662	0.00586494
tanespimycin	0.22895161	0.00613157
AGK-2	0.22654675	0.0067061
BRD-K99584050	0.22060307	0.00833627
myricetin	0.22045285	0.00838166
HBX-41108	0.21521083	0.010109
EX-527	0.21082434	0.01178795

ML203	0.21032853	0.01199232
BRD-K13185470	0.20930658	0.01242337
BRD-K29086754	0.2075499	0.01319611
bosutinib	0.20655789	0.01365079
PF-3758309	0.20404396	0.01486485
I-BET151	0.2019371	0.01595376
AZ-3146	0.20186995	0.01598958
BRD-K70511574	0.20115368	0.01637601
0rciclasine	0.20069436	0.01662807
CI-976	0.19523391	0.01989247
VAF-347	0.19236067	0.02182247
NVP-231	0.19222135	0.02192002
BRD9876	0.19203502	0.02205106
tivozanib	0.19114593	0.02268569
parbendazole	0.19037527	0.02324842
0vitoclax:gemcitabine (1:1 mol/mol)	0.1885341	0.02464156
marinopyrrole A	0.18827548	0.02484287
pazopanib	0.18488686	0.02761347
clofarabine	0.18298741	0.02927879
necrostatin-7	0.1754397	0.03676603
BRD-K07442505	0.17174646	0.04098146
BMS-536924	0.17054509	0.04243713
niclosamide	0.1698257	0.04332934
imatinib	0.16923782	0.04407002
RO4929097	0.16796122	0.04571492
cytarabine hydrochloride	0.1659456	0.04841603
tanespimycin:gemcitabine (1:1 mol/mol)	0.16448969	0.05044847
3-Cl-AHPC	0.16361069	0.05170944
BRD-K71935468	0.16108775	0.05547435
SNX-2112:bortezomib (250:1 mol/mol)	0.15896954	0.05880721
FQI-2	0.15884817	0.05900306
BRD-K88742110	0.15551334	0.06459716
docetaxel:tanespimycin (2:1 mol/mol)	0.14956539	0.07564543
ISOX:bortezomib (250:1 mol/mol)	0.14935196	0.07606861
bortezomib	0.14933672	0.07609888
GDC-0879	0.14867441	0.07742464
topotecan	0.14849393	0.07778912
decitabine	0.14659393	0.08171064
OSI-930	0.14599316	0.08298308
Merck60	0.14488658	0.0853684
BRD9876:MK-1775 (4:1 mol/mol)	0.14242239	0.09087722
Compound 23 citrate	0.13603429	0.10647952
bafilomycin A1	0.13326687	0.11385917
decitabine:carboplatin (1:1 mol/mol)	0.13289719	0.1148744
SNS-032	0.1328253	0.11507264
doxorubicin:0vitoclax (2:1 mol/mol)	0.13254752	0.11584117
PF-573228	0.13246968	0.11605723
fluvastatin	0.13219944	0.11680979
AZD8055	0.13069585	0.12106646
LBH-589	0.13063456	0.12124248
PL-DI	0.12729338	0.13114128
dabrafenib	0.1265546	0.13341151
elocalcitol	0.12644148	0.13376176
Compound 7d-cis	0.12599267	0.13515831
BRD-K48477130	0.12586046	0.13557183
ciclopirox	0.12464655	0.13941392
vincristine	0.12182698	0.14865741
AZD7762	0.12178605	0.14879489
valdecoxib	0.12059776	0.15282913
omacetaxine mepesucci0te	0.11980132	0.15557876
doxorubicin	0.11855785	0.15994575
SR8278	0.11816024	0.16136129

BI-2536	0.11485848	0.17347944
apicidin	0.11297326	0.18069359
SNX-2112	0.11248498	0.1825975
ML239	0.11226449	0.18346207
BRD-K09344309	0.1119606	0.18465853
KHS101	0.1113789	0.18696474
chlorambucil	0.11075053	0.18947949
palmostatin B	0.10955874	0.19431672
BRD-K03536150	0.1086009	0.19826887
KX2-391	0.1067071	0.20625338
tacedi0line	0.10623912	0.20826153
phloretin	0.1051587	0.21295127
nutlin-3	0.10281823	0.22336798
StemRegenin 1	0.10256173	0.22453111
BRD9647	0.10078998	0.2326825
piperlongumine:MST-312 (1:1 mol/mol)	0.10055576	0.23377543
MK-2206	0.09928606	0.23976284
manumycin A	0.0971797	0.24992986
vorinostat	0.09676713	0.25195563
NVP-ADW742	0.09204117	0.27596997
nilotinib	0.09120725	0.28036256
gossypol	0.0856257	0.31096863
SR-II-138A	0.08522452	0.31324944
silde0fil	0.08521597	0.31329818
axitinib	0.08454882	0.31711554
ISOX	0.08345878	0.32341728
JQ-1:carboplatin (1:1 mol/mol)	0.08317285	0.32508356
alvocidib	0.08279625	0.32728665
BMS-754807	0.08257189	0.32860371
968	0.08182043	0.33303969
AT13387	0.0816929	0.33379626
BRD-K66532283	0.0813798	0.3356585
tosedostat	0.0809921	0.33797348
WP1130	0.08044764	0.34124167
Ko-143	0.0799458	0.34427166
Ki8751	0.0789186	0.35052649
NSC95397	0.07505952	0.37465646
tivantinib	0.07136387	0.39869235
A-804598	0.06991467	0.4083629
BRD4132	0.06976433	0.40937398
NSC23766	0.06940391	0.41180385
barasertib	0.06764152	0.42380701
quizartinib	0.06761627	0.42398039
BRD-K92856060	0.06620685	0.43372588
JQ-1	0.06595656	0.4354698
BRD-A86708339	0.06532989	0.43985371
PX-12	0.06437398	0.44658887
KU-60019	0.06352037	0.45265194
BRD-K01737880	0.06349611	0.45282494
cyanoquinoline 11	0.06224707	0.46178098
pandacostat	0.0617987	0.46501967
LY-2183240	0.0579773	0.49312335
SMER-3	0.05701902	0.50030931
curcumin	0.05482973	0.51693031
KU-55933	0.05117205	0.54531717
QS-11	0.04892218	0.56315025
bardoxolone methyl	0.04695034	0.5790048
GSK-J4	0.04658231	0.58198678
austocystin D	0.04566937	0.58941431
nelarabine	0.04457704	0.59835778
CIL55A	0.04438155	0.59996468
BRD-K11533227	0.04275612	0.61340026

pifithrin-mu	0.04245373	0.61591414
SB-525334	0.04093144	0.6286371
ML006	0.03997718	0.63666886
Repligen 136	0.03441438	0.68430878
BRD-A71883111	0.03279179	0.69845288
LE-135	0.03187779	0.70646647
KW-2449	0.0310021	0.71417446
BRD-K16147474	0.02953614	0.72714262
zebularine	0.02875977	0.73404224
GSK461364	0.02800643	0.74075763
dacarbazine	0.0277609	0.74295052
AM-580	0.02766495	0.74380801
mitomycin	0.02636605	0.75544724
avrainvillamide	0.02509684	0.76687368
sotrastaurin	0.02235203	0.79175377
selumetinib:decitabine (4:1 mol/mol)	0.02225535	0.79263413
regorafenib	0.02196814	0.79525097
CHIR-99021	0.02004675	0.81281435
lomeguatrib	0.01929101	0.8197486
YM-155	0.01806885	0.83099195
tacrolimus	0.01690528	0.84172825
methylstat	0.01637653	0.84661673
belinostat	0.0162948	0.84737289
0kiterpiosin	0.01596379	0.85043677
isonicotinohydroxamic acid	0.01577008	0.85223087
NSC48300	0.01466719	0.86245944
ABT-199	0.014602	0.86306477
carboplatin:etoposide (40:17 mol/mol)	0.01259835	0.88170696
KU 0060648	0.0124175	0.88339297
BRD-K52037352	0.01231165	0.88438009
KU-0063794	0.01149989	0.8919555
BRD-K51490254	0.01097789	0.89683205
HLI 373	0.01027181	0.90343436
serdemetan:SCH-529074 (1:1 mol/mol)	0.00921457	0.91333254
TG-100-115	0.00827598	0.92213103
leptomycin B	0.00703494	0.93377917
PAC-1	0.00590123	0.94443219
SU11274	0.00512891	0.95169515
oligomycin A	0.00466304	0.95607815
tanespimycin:bortezomib (250:1 mol/mol)	0.00285274	0.97312122
brefeldin A	0.00220673	0.97920645
YL54	0.00165159	0.98443666
GANT-61	0.00132653	0.98749949
SB-743921	0.00096207	0.99093377
tamoxifen	0.00072043	0.99321084
BRD-K90370028	0.00056252	0.99469894
BRD-K33514849	-0.000507	0.99522211
BRD-K63431240	-0.0006913	0.9934855
ML311	-0.0023089	0.97824437
trametinib	-0.0026215	0.97529945
teniposide	-0.0051941	0.95108152
BRD1835	-0.0054333	0.94883214
paclitaxel	-0.0063039	0.94064705
WZ4002	-0.0069766	0.93432697
di0ciclib	-0.0082402	0.9224666
BRD-K34099515	-0.0093831	0.91175359
ceranib-2	-0.0105803	0.90054885
simvastatin	-0.0124691	0.88291237
masitinib	-0.0130816	0.8772046
SN-38	-0.014172	0.86705944
BRD6340	-0.0141898	0.86689409
UNC0638	-0.0145507	0.86354089

PF-184	-0.0149878	0.85948345
KPT185	-0.0152132	0.85739252
CHM-1	-0.0156611	0.85324031
BRD-M00053801	-0.0157206	0.8526891
YK 4-279	-0.0160691	0.84946143
selumetinib:BRD-A02303741 (4:1 mol/mol)	-0.018012	0.83151621
piperlongumine	-0.018292	0.82893645
OSI-027	-0.0183123	0.82874993
BRD-K27188169:0vitoclax (2:1 mol/mol)	-0.0186377	0.8257546
SZ4TA2	-0.0187323	0.82488428
CIL41	-0.0206839	0.80697931
rigosertib	-0.0230155	0.7857193
IC-87114	-0.0241818	0.77514268
trifluoperazine	-0.0246721	0.7707089
BRD-K55116708	-0.0267002	0.75244761
epigallocatechin-3-monogallate	-0.0268308	0.75127663
crizotinib	-0.0286037	0.73543214
ML320	-0.0287085	0.73449832
BRD-K50799972	-0.0305026	0.71858457
BRD-K61166597	-0.030584	0.71786485
B02	-0.0309912	0.7142704
GSK525762A	-0.0314802	0.70996289
ML050	-0.0322102	0.70354846
salermide	-0.0324778	0.7012021
CID-5951923	-0.0340097	0.68782644
SCH-79797	-0.0349706	0.67948493
triazolothiadiazine	-0.0356906	0.67325991
SB-225002	-0.0376047	0.65681991
decitabine:0vitoclax (2:1 mol/mol)	-0.0379815	0.65360229
CIL56	-0.0386	0.64833491
neopeltolide	-0.0395913	0.63992884
BRD-K34222889	-0.0407443	0.63020896
selumetinib:MK-2206 (8:1 mol/mol)	-0.0418321	0.62109629
isoliquiritigenin	-0.0420537	0.61924689
alisertib	-0.0434951	0.60727553
RG-108	-0.044108	0.60221689
SCH-529074	-0.045223	0.59306123
ifosfamide	-0.0472534	0.57655461
erlotinib	-0.0484128	0.56722582
temozolomide	-0.0490243	0.56233514
BRD-K30748066	-0.0499777	0.55474954
NSC 74859	-0.0505705	0.55005777
staurosporine	-0.0508275	0.54803001
selumetinib:JQ-1 (4:1 mol/mol)	-0.0534468	0.52757361
entinostat	-0.0534964	0.52719012
dexamethasone	-0.0537123	0.52552165
tipifarnib-P1	-0.0548136	0.51705384
ibrutinib	-0.0577306	0.49496835
momelotinib	-0.0600771	0.47757078
ML031	-0.061143	0.46977829
BMS-195614	-0.0616831	0.46585673
BMS-270394	-0.0625003	0.45995756
BRD-K29313308	-0.0629212	0.4569349
blebbistatin	-0.0639338	0.44970963
BRD-A94377914	-0.0642041	0.44779167
cyclophosphamide	-0.0648014	0.4435703
QW-BI-011	-0.0649606	0.44244861
selumetinib:vorinostat (8:1 mol/mol)	-0.0658166	0.43644701
necrostatin-1	-0.0669326	0.42869198
ML162	-0.0670551	0.42784563
selumetinib:PLX-4032 (8:1 mol/mol)	-0.0671367	0.42728176
AT7867	-0.0682481	0.41965316

AZD6482	-0.0718825	0.39526481
fingolimod	-0.0739359	0.38186902
GW-405833	-0.075378	0.37262731
PLX-4720	-0.0760991	0.36805854
MST-312	-0.0807385	0.33949347
pluripotin	-0.0808665	0.33872593
vorinostat:0vitoclax (4:1 mol/mol)	-0.0818643	0.33277978
COL-3	-0.0827266	0.32769521
selumetinib	-0.0835529	0.32286972
BRD-K17060750	-0.0840612	0.3199249
CD-437	-0.0850435	0.31428239
bleomycin A2	-0.0858355	0.30978041
CBB-1007	-0.0872937	0.30160198
prochlorperazine	-0.0877169	0.29925562
AZD4547	-0.0886583	0.29407921
itraco0zole	-0.0900409	0.28658482
DBeQ	-0.0921594	0.27535081
etoposide	-0.0929935	0.27101057
TPCA-1	-0.0935972	0.26789823
BRD-A02303741:0vitoclax (2:1 mol/mol)	-0.0938066	0.2668244
neuro0l differentiation inducer III	-0.095463	0.25843348
LRRK2-IN-1	-0.0965933	0.25281237
BRD-K41597374	-0.0975005	0.2483625
BRD-K66453893	-0.0978987	0.24642626
isoevodiamine	-0.0993667	0.2393793
ETP-46464	-0.1003494	0.23474148
Mdivi-1	-0.1023269	0.22559959
JQ-1:vorinostat (2:1 mol/mol)	-0.103019	0.22246073
AT-406	-0.1055288	0.21133633
BRD-K55473186	-0.1057415	0.21041237
tigecycline	-0.108196	0.19995706
lenvatinib	-0.1107619	0.18943367
dari0parsin	-0.1146345	0.17432517
PF-543	-0.1154804	0.17114681
0vitoclax:sorafenib (1:1 mol/mol)	-0.1162615	0.16825028
NVP-BEZ235	-0.1192053	0.15766067
AA-COCF3	-0.1201505	0.15436882
tretinoin:carboplatin (2:1 mol/mol)	-0.1204696	0.15326912
sunitinib	-0.1208173	0.15207771
abiraterone	-0.1212347	0.15065677
BRD-K75293299	-0.1248844	0.13865465
PF-750	-0.1252806	0.13739695
fulvestrant	-0.1257656	0.13586904
PLX-4032	-0.126413	0.1338501
GW-843682X	-0.1267189	0.13290412
GSK-3 inhibitor IX	-0.1284759	0.12756948
AC55649	-0.1294708	0.12462257
cimetidine	-0.1308931	0.12050123
WZ8040	-0.1310793	0.11996977
temsirolimus	-0.1315963	0.11850317
JQ-1:MK-0752 (1:1 mol/mol)	-0.1318584	0.11776506
BRD-K35604418	-0.1327053	0.11540409
ML210	-0.1342956	0.11107084
carboplatin:UNC0638 (2:1 mol/mol)	-0.1352306	0.10858286
semagacestat	-0.1353183	0.10835182
BRD8958	-0.1359399	0.10672496
PD318088	-0.1364404	0.10542882
XL765	-0.138019	0.10142208
SKI-II	-0.1380344	0.10138344
obatoclax	-0.1383539	0.10058769
0vitoclax:MST-312 (1:1 mol/mol)	-0.1392842	0.098298
PD 153035	-0.1406998	0.09489332

ruxolitinib	-0.1457128	0.08358226
GSK1059615	-0.1460605	0.08283957
tipifarnib-P2	-0.1461599	0.08262827
tamatinib	-0.148427	0.07792458
BRD-K51831558	-0.1491425	0.07648581
CCT036477	-0.1509297	0.07298515
ABT-737	-0.1520238	0.07090692
Bax channel blocker	-0.1521721	0.07062887
1S,3R-RSL-3	-0.1526143	0.06980537
neratinib	-0.1535044	0.06817122
VX-680	-0.154533	0.06632157
serdemetan	-0.1551257	0.06527472
selumetinib:0vitoclax (8:1 mol/mol)	-0.1557439	0.06419692
indisulam	-0.1559211	0.06389067
afatinib	-0.1579957	0.06039379
lovastatin	-0.1587283	0.05919697
dasatinib	-0.1594255	0.05807615
sirolimus:bortezomib (250:1 mol/mol)	-0.1600461	0.0570933
tandutinib	-0.1606385	0.05616796
canertinib	-0.1630077	0.05258954
silmitasertib	-0.1634303	0.0519715
myriocin	-0.165053	0.04965385
parthenolide	-0.1669542	0.04704832
CD-1530	-0.1671609	0.04677194
MGCD-265	-0.168437	0.04509599
BRD-K94991378	-0.1698042	0.04335622
BRD-A02303741	-0.1717135	0.04102077
SID 26681509	-0.1720321	0.04064155
erastin	-0.1726362	0.03993055
BIX-01294	-0.172753	0.03979425
vorinostat:carboplatin (1:1 mol/mol)	-0.1735768	0.03884431
oxaliplatin	-0.1741588	0.03818467
PIK-93	-0.1772825	0.03480321
docetaxel	-0.1792974	0.03275913
selumetinib:tretinoin (2:1 mol/mol)	-0.1811167	0.03100173
BRD-K26531177	-0.1839477	0.02842646
MLN2480	-0.1850567	0.02746854
UNC0638:0vitoclax (1:1 mol/mol)	-0.1907527	0.02297136
alisertib:0vitoclax (2:1 mol/mol)	-0.1908471	0.0229025
GDC-0941	-0.1913562	0.02253417
NVP-TAE684	-0.1916262	0.02234096
tretinoin:0vitoclax (4:1 mol/mol)	-0.1942112	0.02056185
0vitoclax	-0.1956689	0.01961355
cerulenin	-0.1969645	0.01880263
selumetinib:UNC0638 (4:1 mol/mol)	-0.2015812	0.01614439
0vitoclax:piperlongumine (1:1 mol/mol)	-0.2054554	0.01417211
R428	-0.2060051	0.01391005
biri0pant	-0.2062935	0.01377428
BRD-K02492147	-0.2071732	0.01336718
AZD1480	-0.2097512	0.01223418
lapatinib	-0.2111103	0.01167149
PRIMA-1	-0.211619	0.01146678
SRT-1720	-0.2117352	0.01142045
carboplatin	-0.2139892	0.01055399
JQ-1:0vitoclax (2:1 mol/mol)	-0.2147255	0.01028376
cytochalasin B	-0.2151807	0.01011976
avicin D	-0.2167103	0.00958545
PDMP	-0.2192392	0.00875649
tretinoin	-0.2199332	0.0085404
ELCPK	-0.2208147	0.0082727
0vitoclax:pluripotin (1:1 mol/mol)	-0.2208264	0.00826919
BIBR-1532	-0.220861	0.00825884

RAF265	-0.2217823	0.00798743
thalidomide	-0.2225137	0.00777761
JW-480	-0.2233395	0.00754662
BRD-K33199242	-0.2237119	0.00744445
BRD-K13999467	-0.223744	0.00743569
purmorphamine	-0.2257206	0.00691424
tubastatin A	-0.2286703	0.00619642
ML258	-0.2290974	0.0060982
nintedanib	-0.230084	0.00587655
cucurbitacin I	-0.2303723	0.00581315
PRIMA-1-Met	-0.2320343	0.00545943
olaparib	-0.2322807	0.00540866
Compound 1541A	-0.2375362	0.00442154
0vitoclax:PLX-4032 (1:1 mol/mol)	-0.2381821	0.00431211
PYR-41	-0.2382122	0.00430707
JQ-1:UNC0638 (2:1 mol/mol)	-0.2401183	0.00399847
NVP-BSK805	-0.2410741	0.00385133
foretinib	-0.2425775	0.00362971
N9-isopropylolomoucine	-0.2453409	0.00325208
TGX-221	-0.2455484	0.00322521
selumetinib:GDC-0941 (4:1 mol/mol)	-0.2457638	0.00319752
BRD-K28456706	-0.2478926	0.00293522
BRD-K48334597	-0.2487819	0.00283152
HC-067047	-0.2494724	0.0027533
TG-101348	-0.2501735	0.00267587
PI-103	-0.2505628	0.00263373
BCL-LZH-4	-0.2534249	0.00234193
BRD-K24690302	-0.2545168	0.00223855
CAL-101	-0.2572557	0.00199719
CAY10594	-0.2590653	0.00185095
PRL-3 inhibitor I	-0.2659281	0.00138055
MG-132	-0.2709818	0.00110695
cabozantinib	-0.2743739	0.00095217
sirolimus	-0.2764269	0.00086839
MK-1775	-0.2773121	0.0008344
NSC632839	-0.2799808	0.00073918
BRD-K09587429	-0.2813013	0.00069585
Ch-55	-0.2822243	0.00066697
BRD1812	-0.2825562	0.00065685
BRD-K27986637	-0.2845158	0.00059996
SB-431542	-0.2904923	0.00045326
ZSTK474	-0.2911597	0.00043912
BRD-K96970199	-0.2914566	0.00043297
BRD-K42260513	-0.2950354	0.00036475
gefitinib	-0.3036813	0.00023883
BRD-K86535717	-0.3059544	0.0002132
ML334 diastereomer	-0.3066236	0.00020615
FSC231	-0.3068103	0.00020423
BRD-K85133207	-0.3101459	0.00017251
pifithrin-alpha	-0.3111945	0.00016353
veliparib	-0.3129616	0.00014937
WAY-362450	-0.3133415	0.00014648
BYL-719	-0.3156147	0.00013024
BRD-K80183349	-0.3196672	0.00010538
LY-2157299	-0.3232028	8.74E-05
16-beta-bromoandrosterone	-0.323766	8.48E-05
istradefylline	-0.3272629	7.03E-05
GSK2636771	-0.3273628	6.99E-05
0vitoclax:biri0pant (1:1 mol/mol)	-0.3279688	6.76E-05
cediranib	-0.3282061	6.68E-05
MK-0752	-0.3307135	5.83E-05
BRD-A05715709	-0.3327406	5.21E-05

JW-74	-0.334839	4.64E-05
BIRB-796	-0.3357639	4.41E-05
sitagliptin	-0.3485478	2.13E-05
MI-1	-0.3485911	2.12E-05
triptolide	-0.3489987	2.07E-05
BRD-K19103580	-0.3508084	1.87E-05
C6-ceramide	-0.3519096	1.75E-05
SGX-523	-0.3530387	1.64E-05
salermide:PLX-4032 (12:1 mol/mol)	-0.3626261	9.21E-06
crizotinib:PLX-4032 (2:1 mol/mol)	-0.3628368	9.09E-06
BRD-K04800985	-0.3635448	8.71E-06
NPC-26	-0.3640318	8.45E-06
BRD-K27188169	-0.3665333	7.25E-06
ML083	-0.3682388	6.52E-06
saracatinib	-0.3717155	5.25E-06
L-685458	-0.3787289	3.36E-06
erlotinib:PLX-4032 (2:1 mol/mol)	-0.3832858	2.50E-06
JW-55	-0.3842399	2.35E-06
vandetanib	-0.3865323	2.02E-06
CIL55	-0.3882005	1.81E-06
BRD8899	-0.3889544	1.72E-06
BRD-K37390332	-0.3956243	1.10E-06
BRD-K44224150	-0.3979656	9.37E-07
SR1001	-0.3981784	9.23E-07
compound 1B	-0.4029139	6.65E-07
BRD-K99006945	-0.4055665	5.53E-07
O-6-benzylguanine	-0.405635	5.50E-07
importazole	-0.4073877	4.86E-07
BRD-K45681478	-0.4185326	2.18E-07
BRD-K20514654	-0.4214896	1.75E-07
BEC	-0.4252636	1.32E-07
IU1	-0.4264365	1.21E-07
BRD-K96431673	-0.4291322	9.90E-08
fumonisin B1	-0.434466	6.58E-08
vorapaxar	-0.4351299	6.25E-08
BRD-K41334119	-0.4378822	5.05E-08
GSK4112	-0.4525721	1.56E-08
ciclosporin	-0.4553575	1.24E-08
BRD-K78574327	-0.4556075	1.22E-08
UNC0321	-0.4672777	4.56E-09
NSC30930	-0.4727798	2.83E-09
PF-4800567 hydrochloride	-0.4796229	1.55E-09
BRD-K02251932	-0.4842575	1.02E-09
IPR-456	-0.4893739	6.41E-10
BRD-K30019337	-0.4939581	4.19E-10
BRD-K49290616	-0.497229	3.08E-10
ML312	-0.4985835	2.71E-10
BRD-K84807411	-0.5011423	2.12E-10
BRD-K03911514	-0.503132	1.76E-10
BRD-K34485477	-0.50645	1.27E-10
VU0155056	-0.5125204	7.02E-11
FGIN-1-27	-0.5142558	5.91E-11
BRD-K14844214	-0.5230701	2.42E-11
BRD-K27224038	-0.560277	4.17E-13
BRD-K71781559	-0.6972612	5.48E-22
